# The Fate of Leydig Cells in Men with Spermatogenic Failure

**DOI:** 10.3390/life12040570

**Published:** 2022-04-12

**Authors:** Daria Adamczewska, Jolanta Słowikowska-Hilczer, Renata Walczak-Jędrzejowska

**Affiliations:** Department of Andrology and Reproductive Endocrinology, Medical University of Lodz, 92-213 Lodz, Poland; daria.adamczewska@umed.lodz.pl (D.A.); jolanta.slowikowska-hilczer@umed.lodz.pl (J.S.-H.)

**Keywords:** estradiol, Leydig cells, male infertility, non-obstructive azoospermia, spermatogenic failure, testicular dysgenesis syndrome, testosterone

## Abstract

The steroidogenic cells in the testicle, Leydig cells, located in the interstitial compartment, play a vital role in male reproductive tract development, maintenance of proper spermatogenesis, and overall male reproductive function. Therefore, their dysfunction can lead to all sorts of testicular pathologies. Spermatogenesis failure, manifested as azoospermia, is often associated with defective Leydig cell activity. Spermatogenic failure is the most severe form of male infertility, caused by disorders of the testicular parenchyma or testicular hormone imbalance. This review covers current progress in knowledge on Leydig cells origin, structure, and function, and focuses on recent advances in understanding how Leydig cells contribute to the impairment of spermatogenesis.

## 1. Introduction

The testicle is the primary organ responsible for maintaining male fertility and the hormonal state. Not only are these functions fundamental for the upkeep of male sexual characteristics, but they are also fundamental for fertility and species preservation [1]. The main cells engaged in maintaining these functions are Leydig cells (LCs), which reside in the testicular intertubular space, and Sertoli cells (SCs), which dwell inside seminiferous tubules [2]. SCs are responsible for supporting the regulation of the spermatogenesis process [3] and the supply of nutrients to germ cells [1]. LCs, on the other hand, are in charge of the synthesis and secretion of androgens, necessary for the masculinization of male fetuses, together with the initiation and maintenance of spermatogenesis [4,5]. In addition, LCs produce proteins with endogenous and xenotoxic metabolic functions, which also reduce oxidative stress, which thus protects the testicle from toxins [6,7].

Spermatogenic failure, manifested as azoospermia, is frequently linked to impaired LC activity [8,9,10,11,12,13]. Such deteriorated functioning is mainly manifested as histological and hormonal alterations in the testis compartment. These changes worsen with the severity of testis dysfunction [9,14]. Variations in androgen levels may be significant signs of either causal or developmental disturbances of both the androgen-producing and spermatogenic compartments of the human testicle [14]. Proper masculinization requires adequate testosterone (T) production early in fetal development, specifically in the masculinization programming window (MPW). In rats, experimentally-induced testicle disorders occur in response to disruption of fetal androgen production/action during MPW [15]. Subsequently, it has been proposed that there is a linkage between male reproductive disorders that occur at birth and those that emerge in adolescence [16]. To understand early susceptibilities to disturbance, we must first understand T production, including the cells involved.

The present review discusses recent insights from studies of the formation and function of LCs, as well as recent breakthroughs in understanding LCs contribution to diminished testicular function in spermatogenic failure.

## 2. The Development and Function of Leydig Cells

LCs are one of the most important cells in the interstitial tissue of the testicles. They play crucial roles in the development and function of the male reproductive organs [17]. The androgens produced by LCs are required for male genital differentiation and masculinization. Mammals, including humans, have two types of LCs, viz. fetal Leydig cells (FLCs) and adult Leydig cells (ALCs), which are present in the fetal and adult testes, respectively [18,19,20,21]. Some authors distinguish an additional neonatal Leydig cell (NLC) population in humans [21,22,23,24].

### 2.1. Fetal Leydig Cells

Although the origin of FLCs is unknown, several hypotheses have emerged. In rodents, FLCs are found in the testicular interstitium on gestational days 11–12 and are assumed to derive from numerous embryonic tissues, including the coelomic epithelium, migrating neural crest cells and mesonephric or epithelial populations of the neighboring mesonephros, early after testis determination. Evidence also suggests that FLCs and adrenal cortex cells share a common ancestor. However, none of the suggested progenitor pools have been shown to directly give rise to FLCs [18,25,26]. FLC differentiation is thought to be influenced by SC activity, and it has been demonstrated that SC products like desert hedgehog (DHH) or platelet derived growth factor (PDGFA) are essential for FLC development [26]. FLC numbers increase significantly during embryonic development, although these cells are not mitotically active. This means that new cells must arise as a result of progenitor cell differentiation and not the division of existing cells [25,27]. Human FLCs reach their peak differentiation at week 14, and maturation at week 18 of gestation. Later, up to the moment of full-term birth, FLC involution occurs [28]. So far, no information is available on the circulating factors that cause the degeneration of human FLCs. It is suspected that such signaling factors as anti-Müllerian hormone (AMH), gonadotropin-releasing hormone (GnRH), and transforming growth factor (TGF) may be responsible for the degeneration of FLCs in rodents [29,30]; however, their role in the regression of human FLCs is unknown [31].

FLCs are round to oval in form, and densely packed with lipid droplets. In the interstitium of prenatal rats, they tend to form clusters and are surrounded by a basement membrane containing collagen and laminin-based extracellular matrix. A similar discovery was made in humans, providing ultrastructural evidence for the basement membrane surrounding FLCs [32].

FLCs mainly produce androgen hormones that contribute to the development of male internal and external genitalia [18]. Testosterone (T), the most potent androgen in mammals, is derived from cholesterol through a chain of events mediated by a group of steroidogenic enzymes. Most of these enzymes are expressed in FLCs, except for one enzyme which mediates the final response to T production. Consequently, the major androgen produced by FLC is androstenedione, not T. Androstenedione is converted to T by fetal SCs [33,34]. In humans, between 8 and 20 weeks of pregnancy, T determines the formation of male internal genitalia from Wolffian ducts (also known as the mesonephric ducts) [35,36]. Human chorionic gonadotropin (hCG), secreted by the placenta, is the principal stimulator of T release until week 12 of pregnancy. Following this, luteinizing hormone (LH) receptors are expressed in FLCs, and after 16 weeks, T synthesis is controlled by LH [37]. The development of the external genitals occurs under the influence of dihydrotestosterone (DHT), which is primarily transformed locally from T by the enzyme 5-alpha-reductase [38]. The androgens produced by the FLCs and insulin-like factor 3 (INSL3) also influence the final stage in the development of the male gonad: the descent of the fully formed testicles into the scrotum [39]. The two phases of testicular descent are controlled differently, with the first transabdominal phase being largely dependent on INSL3, and the second inguinoscrotal phase being largely dependent on androgens. INSL3 acts on the relaxin family peptide 2 receptor (RXFP2), also known as leucine-rich repeat-containing G protein-coupled receptor 8 (LGR8), in the gubernacular ligaments that connect the testes to the inguinal region of the abdominal cavity [40,41,42]. INSL3, like T, is produced in a LH-dependent manner and is thought to be a sensitive marker of LC function and differentiation status [43,44]. INSL3 is strongly expressed in the fetal testis, but it is suppressed after birth, only to reappear after puberty [45]. This hormone is believed to have multiple functions in adult males since it operates as a paracrine mediator on reproductive cells as well as an endocrine factor elsewhere. Importantly, INSL3 levels appear to better indicate the functional condition of LCs than T levels [46].

### 2.2. Neonatal Leydig Cells

There is another generation of LCs in primate and human testes that form and vanish in a relatively brief amount of time during the neonatal stage. Although it is currently unknown where this generation of NLCs originate from, the existence of a triphasic pattern of human LCs development is now acknowledged as a working model [21,22,23,24]. The appearance of differentiated NLCs indicates the recruitment of precursors in the neonatal period. Some authors have suggested that the FLC population is involved in this phenomenon [28]. Moreover, the formation of NLCs and their function are thought to be governed by an increase in LH levels caused by hypothalamic–pituitary–gonadal (HPG) axis reactivation during the neonatal period. This is supported by the discovery of the presence of cells isolated from postnatal human testicles; these react more strongly to LH in the first few months of life than in the second and third year, with the simultaneous presence of FLCs [47]. The development of NLCs is associated with a significant increase in the production of androgens, mainly T. The exact physiological function of this short T-outburst by NLCs in humans is not known; however, it has been theorized that it may be important for imprinting different types of cells in androgen-dependent organs, e.g., the brain, to ensure their correct adult response to androgens [31].Over the next few months, the NLCs peak in number and, at the same time, the FLCs regress. Before the first signs of pubertal development appear, the HPG axis becomes inactive again and NLCs undergo involution or dedifferentiation [22]. The following resting phase in LC activity lasts for years until the development of the next, third generation of LCs, i.e., ALCs, begins [48].

### 2.3. Adult Leydig Cells

LC maturation is required for the initiation and maintenance of spermatogenesis, as well as for the promotion of secondary sexual characteristics in men [49]. ALCs arise after birth from a pool of stem cells known as stem Leydig cells (SLCs). The differentiation of LC from tissue stem cells to fully mature LCs with maximum steroidogenic capacity has been well characterized in rats. Adult rat LC differentiation is believed to occur in four consecutive stages; stem cells that differentiate into spindle-shaped precursor Leydig cells (PLCs), round immature Leydig cells (ILCs), and polygonal mature ALCs. These forms were discovered in studies on rats [50,51,52] and similar forms have been described in human testes [21]. The transition from SLCs to PLCs, ILCs, and ultimately ALCs is governed by LH, androgens, and numerous growth factors, including DHH, PDGFA, TGF, leukemia inhibitory factor (LIF), KIT ligand (KITLG), insulin-like growth factor 1 (IGF1), and fibroblast growth factor 2 (FGF2) [48]. ALCs, like other differentiated cells, have very little mitotic activity, and any recovery in the LC population depends on recruitment from tissue stem cells and/or LCs precursor pools [53]. SLCs can be found in the early interstitial compartment of the testicles shortly after birth. Additionally, spindle-shaped cells serving as precursors to ALCs have been found in the peritubular and perivascular regions of adult testes [54,55]. These cells were found to be able to self-renew indefinitely, or to specialize and eventually create T, just like the stem cells of the neonatal testis [20]. Based on a previous study on the induction of hypothyroidism in neonatal rats, it has been hypothesized that an increase in the number of LCs in the testes of such animals during adulthood occurs due to a simultaneous rise in the number of SCs [56]. However, in a study using a GnRH antagonist in neonatal rats, despite a significant decrease in SC numbers, no substantial reduction in LC nuclear volume or number per testis was observed in any of the treatment groups in adulthood. This study also suggested that gonadotrophins, androgens, or estrogens did not significantly affect the LC number of treated rats during the first two weeks after birth. This could mean that the ultimate ALC number can be determined before birth [57].

### 2.4. The Relationship between Fetal and Adult Leydig Cells

**Figure 1:**
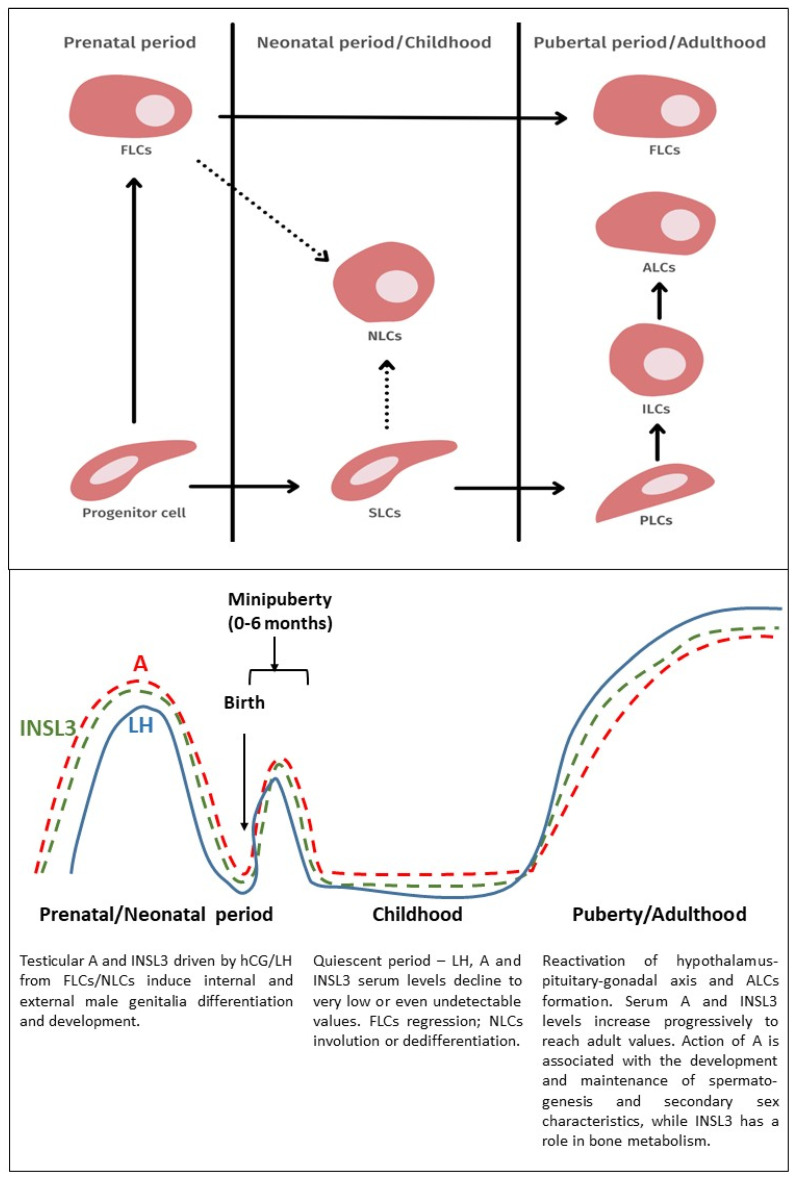
(**Upper panel**): Proposed model of Leydig cell lineage development. In the fetal testis, there is a shared Leydig cell progenitor pool that gives birth to both fetal Leydig cells (FLCs) and adult Leydig cells (ALCs). FLCs form in the fetal testis’ interstitial area. In the neonatal testis progenitor cells develop into stem Leydig cells (SLCs), which is the initial step in ALC subgroup differentiation. The dotted arrows show the hypothetical origin of neonatal Leydig cells (NLCs). NLCs are supposedly derived from either non-degraded FLCs or newly formed SLCs. NLCs slowly regress after the first postnatal year. During puberty, SLCs develop into mature cells via stages of newly progenitor Leydig cells (PLCs), immature Leydig cells (ILCs), and ALCs. A fraction of FLCs remains in the adult testis and accounts for approximately 10% of the total Leydig cell pool. However, it is still unknown how each progenitor is destined to become FLCs or ALCs. (**Lower panel**): Serum levels of luteinizing hormone (LH) secreted by pituitary and androgens (A) and insulin-like factor 3 (INSL3) produced by Leydig cells at different stages of their development. During the prenatal period fetal LH surges at mid-gestation, then declines and is low or undetectable in cord blood. The pattern of androgens and INSL3 concentrations follow that of LH. At birth, LH, androgens, and INSL3 levels are low and increase during the first weeks and months (minipuberty) to reach peak levels during the third month of life and then gradually decline and remain low at childhood. At puberty, LH, androgens, and INSL3 increase to reach the levels characteristic for adulthood. The main role of androgens and INSL3 in prenatal/neonatal period is induction of internal and external male genitalia differentiation and development. At puberty and then in adulthood, the main androgen action is associated with the development and maintenance of secondary sex characteristics and spermatogenesis, while INSL3 exerts a role in bone metabolism.

The conjunction between FLCs and ALCs remains unclear. The first reports indicated that the postpartum FLCs niche completely regressed and was replaced by ALCs. However, this thesis has recently been disproved. The presence of FLCs can be found at the peak of hormone secretion, immediately after birth, in the so-called “mini-puberty” phase [58]. Then comes a period of quiescence for many years. During this period, most FLCs disappear or becomes morphologically unrecognizable [19]. However, some of them will survive and remain present in the mature testis [59,60]. Recent cell line-tracking studies in mice proved that FLCs can be found in the gonads of adult males, and they account for about 10% of the total LC population [61,62]. While these persistent FLCs appear grossly similar to developing ALCs, there are some subtle differences. FLCs have many large droplets of cytoplasmic lipids while ALCs have relatively smaller droplets in fewer numbers [18]. In addition, fetal and adult LCs exhibit different gene expression profiles. Correspondingly, it was hypothesized that LCs found in the fetal testis do not develop into adult cells. Instead, these two cell types arise as two separate lines with different functions and cell origins [48,63,64,65]. It is now believed that FLC and ALC progenitor cells are present in the fetal testes [65,66] and some researchers claim that FLCs and ALCs have the same set of precursors in the fetal testes [51,60]. While conclusive evidence is still lacking, recent research indicates the existence of a single ancestor that remains dormant until puberty and then forms ALCs. In addition, the origin of FLCs is currently not exactly known [67]. Final identification of the FLC source population is necessary to fully understand the genesis and nature of these cells. Currently, most of the evidence for potential FLC source populations is largely based on expression patterns of the characteristic factors that may define the lineage of the FLCs. The existence and function of FLCs, on the other hand, have recently been demonstrated to be significant in the creation and function of ALCs in a mouse model [68]. It is still uncertain if FLCs and ALCs are separate cell populations or share a common stem/progenitor cell lineage; nonetheless, stem ALCs have been found in the human fetal testis [19,69]. Despite this, the lineage interconnection between FLCs and ALCs remains uncertain, and the problem of the sequential evolution of two distinct LCs populations remains an issue [2,70]. Figure 1 presents a proposed model of LCs development and their function.

## 3. The Contribution of Leydig Cells to Spermatogenesis

Spermatogenesis is a complex process regulated by endocrine and testicular paracrine/autocrine factors. SCs play a major role in this process; however, new research has shown that other cell types play an equally important role in the development of reproductive cells [71]. Many studies indicate that many of the cells existing in the interstitial compartment of the human testes are involved in the regulation of the spermatogonial stem cells (SSCs) niche. These cell types are important for fetal testicular morphogenesis [72,73,74] and for spermatogenesis stimulation in adulthood to ensure male fertility [75]. The LCs drive spermatogenesis through the secretion of androgens, other hormones, cytokines, growth factors, transcription factors, and receptors associated with LCs [76]

### 3.1. Steroidogenesis

**Figure 2:**
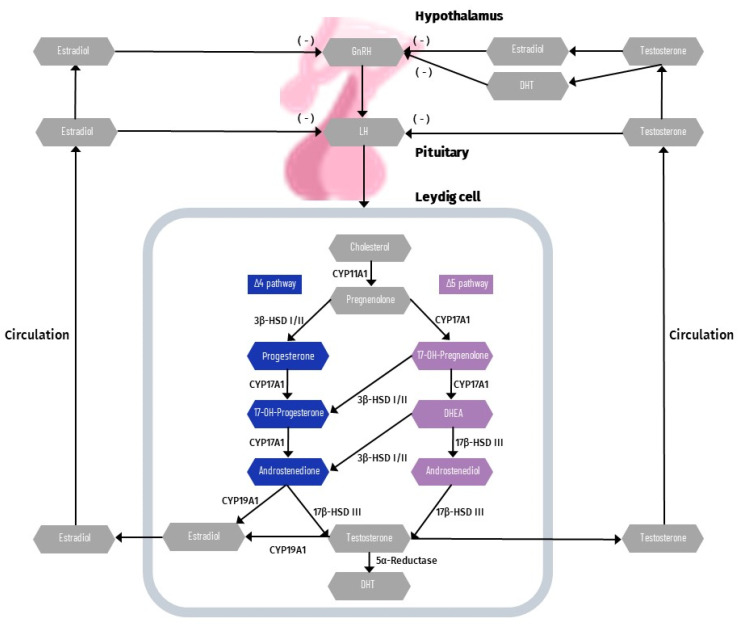
Schematic diagram of regulatory axis of testicular steroidogenesis. Sex steroids (predominantly testosterone) are produced upon central gonadotropin-releasing hormone (GnRH)—luteinizing hormone (LH) stimulation. LH binds to LH receptors on Leydig cells in the testicle and activates the pathway for the synthesis of steroid hormones from cholesterol. Different pathways variants are available after conversion to pregnenolone (Δ4 and Δ5). Normal, adult testicular steroidogenesis in men follows steroidal pathway Δ5, with a little amount of testosterone generated via the Δ4 pathway. Small amount of testosterone and androstendione are converted in the Leydig cells to estrogens by enzyme aromatase (CYP19A1) and to dihydrotestosterone (DHT) by 5α-reductase. Testosterone and estradiol act locally in testicle to regulate its function (e.g., spermatogenesis) or are released to blood circulation. Circulating sex steroids form a negative feedback loop to inhibit the secretion of GnRH and LH.

Steroidogenesis is the biosynthetic process of converting cholesterol into steroid hormones, including T [77]. Although they are synthesized mainly in the gonads, steroid hormones are also produced in the adrenal glands. In the adult testis, production of T in LCs is dependent on the pituitary gland’s pulsatile release of LH into the peripheral circulation. In LC steroidogenesis, LH have two important roles: maintaining appropriate levels of steroidogenic enzymes and mobilizing and transporting cholesterol to the inner mitochondrial membrane. LH induces adenosine triphosphate (ATP) conversion to cyclic adenosine monophosphate (cAMP), which then catalyzes the production of protein kinase A, which is required for the transfer of cholesterol from the cytoplasmic pool to mitochondria. The peripheral benzodiazepine receptor (PBR) and the steroidogenic acute regulatory protein (StAR) transport cholesterol from the outside to the inner mitochondrial membrane [78,79]. On the matrix side of the inner mitochondrial membrane, cholesterol is converted to pregnenolone by the C27 cholesterol side-chain cleavage cytochrome P450 enzyme (CYP11A1). Then, in the smooth endoplasmic reticulum, pregnenolone is transformed to T via three steroidogenic enzymes: 3-hydroxysteroid dehydrogenase (3β-HSD), 17-hydroxylase/17,20-lyase P450 (CYP17A1), and 17-hydroxysteroid dehydrogenase type III (17β-HSD III) [77,80,81,82]. T synthesis from cholesterol can take place through several pathways. Normal adult testicular steroidogenesis in humans follows the Δ5 pathway, with a little amount of testosterone generated via the Δ4 pathway [83]. However, in rodents, testicular steroidogenesis primarily follows the Δ4 pathway [84]. Cholesterol in the ∆5 pathway is converted using the following intermediates: pregnenolone, 17-OH-pregnenolone, dehydroepiandrosterone (DHEA), and androstenediol. In contrast, the ∆4 pathway uses androstenedione as the last precursor to T. Androstenedione can be created by the conversion of DHEA as well as pregnenolone to progesterone and then to 17-OH-progesterone [85]. Small amounts of T or androstenedione are converted to estrogens in the testis by aromatase (CYP19A1). Figure 2 presents a schematic diagram of regulatory axis of testicular steroidogenesis.

### 3.2. Testosterone

LCs are the major population of steroidogenic cells in the testicular interstitium and their undisputed contribution to male reproductive functions is the synthesis of T, which is fundamental for the progress of spermatogenesis [86]. This androgen, under the influence of the HPG axis, diffuses into the interstitial space and affects the signaling pathways of male reproductive cells by binding to androgen receptors (AR) [87,88,89,90]. It is known from mouse models that germ cells do not exhibit AR, despite the fact that they require androgens for survival and development [91]. Nevertheless, LCs and other somatic cells express AR, and it is generally assumed that these cell types mediate the testosterone needed for spermatogenesis [92]. T is required in at least four critical processes during spermatogenesis: blood–testis barrier (BTB) maintenance, meiosis, SCs sperm adhesion, and sperm release [93]. Analyses of AR-deficient SCs in rodent testes revealed the occurrence of three major fertility disorders: violation of BTB integrity, which exposes post-meiotic germ cells to autoimmune and cytotoxic agents [94,95]; a blockage of the conversion of round sperm to elongated sperm due to a cell adhesion defect that causes the premature detachment of round spermatids from Sertoli cells [87,96,97,98]; retention and phagocytosis of mature spermatozoa by SCs [97]. Another study involving knockout of AR in mice indicated that T signaling is essential for several types of somatic cells to uphold spermatogenesis and preserve male fertility [99].

### 3.3. Estradiol

Estrogen has been recognized as another critical regulator of spermatogenesis in various animals, including humans [100]. Estrogen receptor 1 (ESR1, also known as ERα), estrogen receptor 2 (ESR2, also known as ERβ), along with G protein-coupled estrogen receptor (GPER), are abundant in the testes [101,102,103]. The SCs are thought to be the first location of estrogen production in the testis, switching to the LCs during neonatal development, when a gonadotropin-regulated aromatase is present. Estrogen acts on cells possessing estrogen receptors (ER), thus inducing spermatogenesis. Human LCs have low ESR1 and ESR2 levels but high GPER levels [101].

The most important estrogen produced in the human testis is estradiol (E2). E2 appears to cause a variety of changes in LCs, depending on the stage of their development. In the fetal and neonatal testes, estradiol acts by blocking the morphogenetic development of LCs from precursor cells [104]. There is further evidence that E2 inhibits LC regeneration in the testes of adult rats administered ethane dimethylsulfonate (EDS) [105]. Moreover, E2 affects T production in LCs. It appears to do so by blocking the activity of many steroidogenic enzymes involved in the synthesis of T [106]. In addition, E2 acts as a germ cell survival factor [107]. Research conducted on aromatase-knockout mice suggests that dietary phytoestrogens may partially prevent abnormal spermatogenesis by reducing germ cell loss [108]. Apart from the survival of reproductive cells, E2 regulates their proliferation and differentiation as well as apoptosis [107,109,110,111,112]. Low sperm count and poor sperm function may be caused by ER mutations [113]. ER inhibitors cause atrophic seminiferous tubules and impaired spermatogenesis in mice [114]. These results, together with those demonstrating progressive disruption of spermatogenesis in aromatase knockout mice [115], indicate the importance of estrogens for the normal function of the adult testis.

### 3.4. Other Factors

Numerous studies have shown that LCs regulate spermatogenesis, not only through the action of T, but also through the secretion of other factors such as growth factors, cytokines, and other hormones [31,59].

The key mitogen involved in the development of many critical for spermatogenesis cells is IGF1 [116,117]. IGF1 promotes proliferation in various types of testicular cells including LCs, SCs, differentiated spermatogonia, and SSCs. Analyses of individual RNA-Seq cells from mouse and human testes showed that IGF1 is mainly expressed by interstitial cells, including LCs. Meanwhile, IGF1r, which encodes the IGF1 receptor (IGF1R), has been detected primarily on spermatogonia among germ cells [118,119,120]. Moreover, studies on the loss of IGF1 signaling in LCs have shown that it has a very detrimental effect on male fertility. According to a detailed developmental analysis in mice with loss of Igf1r, FLC function was normal, but ALC maturation and steroidogenic activity were impaired due to an accumulation of PLCs. Igf1r-deficent mice were infertile, with decreased testicular weight and poor virilization. All of these defects, with the exception of testis size, were absent in SCs-specific knock-out animals, showing that they were caused by Igf1r deletion in LCs [121].

Hormone oxytocin is another substance produced by LCs that plays an important role in the control of spermatogenesis. This is because of the regulation of steroid production in LCs [122]. Mice need oxytocin to produce and release sperm. In studies in mice deficient in oxytocin, the release time of sperm in the seminiferous tubule was significantly delayed, which supports this thesis [123].

The previously described hormone INSL3 is another significant factor in the development of the sperm cells. By administering INSL3 to mice with induced gonadotropin deficiency, it was revealed that it can prevent germ cell loss [124]. Moreover, a correlation has been noted between serum INSL3 levels and persistent spermatogenesis in studies of experimental male hormonal contraception [125]. Consistent with these findings, INSL3 is closely related to spermatogenesis.

During spermatogenesis and steroidogenesis, the cytokine network in the testis also boosts cell growth and differentiation, assisting in the coordination of multifactorial interactions [126]. Studies in rats have found that an important cytokine, macrophage migration inhibitory factor (MIF), is produced by Leydig cells [127]. MIF is secreted into the testicular interstitial fluid by Leydig cells and interacts directly with Sertoli and peritubular cells as a regulating factor for their signaling [127,128]. Another factor, TGFβ, affects LC steroidogenesis, testis development, and spermatogenesis. One of approximately 40 cytokines in the TGF family, TGFβ has been found in the LCs and other somatic cells, as well as the reproductive cells of pigs, rats, mice, hamsters, and humans [129,130,131]. Studies indicate that TGFβ1 and TGFβ3 are abundant in human LCs, as well as their receptors. TGFβ3 has been shown to interact with TGFβ-Receptor II [132] and TGFβ1 with TGFβ-Receptor I [133]. TGFβ1 was not found in the seminiferous tubules. TGFβ3 and TGFβ-Receptor I were found mostly in elongated spermatids, but TGFβ-Receptor II was found only in pachytene spermatocytes and was weak in spermatogonia, spermatids, and SCs. In the SCs, only TGFβ-Receptor II was found. Finding TGFβ isoforms and receptors in the LCs and germ cells of the adult human testis indicates that they have a role in the regulation of spermatogenesis [129]. In addition, due to the partial action of endogenous IL1, Leydig cells activate the production of large amounts of the immunoregulatory cytokine IL6. Both IL1 and IL6 have the ability to control the development of SCs and spermatogenic cells [126,134]. Higher plasma levels of IL6 have been detected in infertile/immunoinfertile men compared to fertile men [135]. The relevance of cytokines and their receptors in testicular cells, as well as their biological origin, is highlighted by these observations. As a result, in male infertility, the concentrations of these autocrine/paracrine factors should be taken into account.

## 4. Spermatogenic Failure and Leydig Cell Function

The World Health Organization estimates that about 9% of couples worldwide suffer from fertility problems, and the male factor is responsible for up to 50% of cases [136,137]. Testicular failure is defined by impairment of the endocrine (T production) and/or exocrine (sperm production) functioning of the testis. Problems during spermatogenesis manifest as decreased or absent spermatozoa production [138,139,140]. Spermatogenic failure, characterized by a complete lack of sperm production, is recognized as the most severe manifestation of male infertility in humans [141]. Global prognosis show that azoospermia, i.e., the complete absence of sperm in semen, affects up to 10% of men struggling with infertility [142,143]. Azoospermia caused by damage to spermatogenesis is classified as non-obstructive azoospermia (NOA) [144].

### 4.1. Non-Obstructive Azoospermia

The etiology of NOA is either lack of sufficient testis stimulation by gonadotrophins or internal testicular damage [145]. Direct causes include genetic abnormalities, congenital anomalies, hypogonadotrophic hypogonadism, post-infectious conditions, oncological treatment, testicular trauma, and the involvement of exogenous factors (Table 1) [137,146,147,148]. The majority of cases, however, are due to unknown causes, which is often referred to as idiopathic NOA [149]. Idiopathic NOA is believed to be caused by genetic defects which have not been fully discovered. Recent global trends attribute an increasingly important role to the contribution of NOA development to environmental factors. Endocrine disrupting chemicals (EDCs) are exogenous compounds that interfere with the stability and control of the male endocrine system, as well as that of the offspring. By crossing the blood–fetal barrier, several EDCs can impair the development of the genitals, thus disrupting embryonic development. Cross-generational inheritance is also possible with some epigenetic processes [150].

### 4.2. Histological Pattern

**Figure 3:**
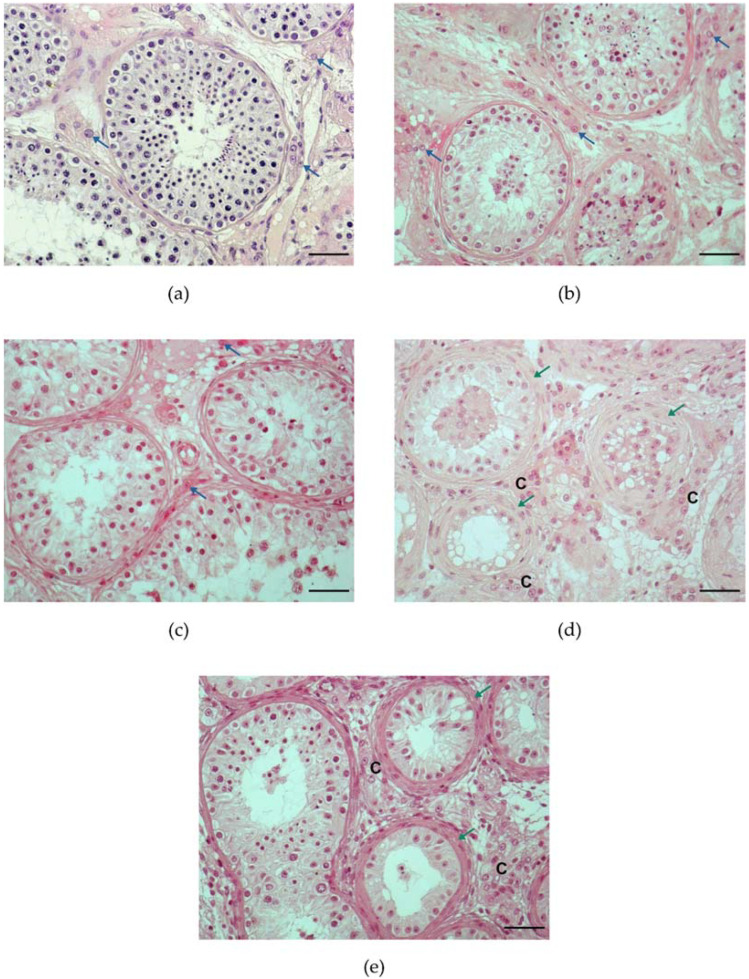
Microphotographs of testicular histology: (**a**) Section of testicular biopsy from men with obstructive azoospermia; normal human testis—well-developed seminiferous tubules with a clear lumen, lined with seminiferous epithelium presenting complete spermatogenesis (qualitatively and quantitatively); a normal number of Leydig cells (LCs) is observed in the intertubular space; LCs are visible as solitary cells or are grouped in small clusters (2–4 cells) (blue arrow); (**b**) Section of testicular biopsy from men with non-obstructive azoospermia presenting hypospermatogenesis—in seminiferous tubules a general decrease in germ cell components is observed; a normal number of LCs is observed in the intertubular space (blue arrow); (**c**) Section of testicular biopsy from men with non-obstructive azoospermia presenting maturation arrest at spermatocyte stage—in seminiferous tubule germ cell development stops during meiosis, a normal number of LCs is observed in the intertubular space (blue arrow); (**d**) Section of testicular biopsy from men with non-obstructive azoospermia presenting Sertoli cell-only syndrome—significantly decreased tubule diameter, increased thickness of basement membrane (green arrow); seminiferous tubules are lined with mature SCs, no germ cells are present; in the intertubular space, an aggregation of LCs is present to form larger clusters (>5 cells) (C). (**e**) Section of testicular biopsy from men with non-obstructive azoospermia presenting mixed atrophy–seminiferous tubules presenting different histological pattern i.e., Sertoli cell-only pattern and hypospermatogenesis; in the intertubular space, an aggregation of LCs can be seen to form larger clusters (>5 cells); note the thickened basement membrane in tubules with Sertoli cell-only pattern (green arrow) in (**d**,**e**). Hematoxylin and eosin staining; Magnification—×200; scale bar, 50 µm.

Men with NOA have different degrees of spermatogenic deterioration [215]. The size of the testes indicates the degree of spermatogenesis; thus, small testes suggest spermatogenesis failure [139]. Some research indicates that the vast majority of NOA patients demonstrate significantly decreased testis volume [12,216]. Normal adult testicular size is determined mainly by germ and SCs, rather than by LCs. However, the LC pool and functioning, as measured by circulating T, are closely related to testicular volume in both pubertal boys [217] and adult males [218]. The histological pattern of the NOA testicles may present a variety of disorders, including hypospermatogenesis (HYPO), spermatogenic cell maturation arrest (MA) at the spermatid, spermatocyte, or spermatogonia level, and absent germ cells in seminiferous tubules (Sertoli cell-only syndrome (SCOS)) [219,220,221,222]. A combination of the aforementioned conditions can be seen in the histological sections of NOA testicular biopsies on occasion, which is termed as “mixed atrophy” (MIX) of seminiferous tubules [223] (Figure 3).

Furthermore, biopsy specimens from males with NOA, particularly those with a history of cryptorchidism and/or numerous foci of testicular microlithiasis, may indicate intratubular germ cell neoplasia in situ (GCNIS) [224,225]. Men with NOA have an increased chance of developing testicular cancer [226]. Hypospadias, cryptorchidism, spermatogenesis impairment, and testicular cancer may have origins in poor prenatal testicular development [227,228,229]. Assessment of the spermatogenesis status shows that NOA patients have a much lower Johnsen score (JS). Semithin sections data revealed a significant loss of diverse spermatogenic cells. There is a clear relationship between the thickness of the lamina propria and the quantity of spermatogenesis in the tubule. Furthermore, prior to spermatogenic failure, the thickness of the lamina propria may increase owing to increased extracellular matrix buildup [230,231]. Progesterone accumulation was seen in the testicular seminiferous tubules’ thicker lamina propria and was linked to spermatogenesis impairment. In the seminiferous tubules of patients with poor spermatogenesis, the progesterone-charged lamina propria is typically thicker. As a result, a change in the metabolic pathway in the human testes is expected, leading to reduced spermatogenesis [230,232]. A study conducted in patients with azoospermia revealed a thickened basement membrane and a reduced diameter of the seminiferous tubules in patients with SCOS. Due to increased deposition of collagen fibrils in the extracellular space, the intact spermatogenic process may be unable to produce sufficient quantities of laminin in the presence of damaged testicular tissue architecture, which might explain the significant decline in seminal laminin in SCOS [233]. In a retrospective study using archival testicular biopsies, AZFa-deleted specimens were found to have a combination of normal to thickened tunica propria, reduced tubular diameter, normal to increased intratubular space, hyperplastic LCs, and SCOS or spermatogenic arrest in a very small number of tubules [152]. Similarly, our group observed a thickened tubular membrane in the testicular tissue of SCOS patients [12].

NOA is usually accompanied by pathological changes in both the tubular and interstitial compartments. Although LCs and vessels make up the majority of interstitial tissues in normal testicles, those with spermatogenic failure have more interstitial tissue and excessive fibrous connective tissue [234]. Semithin sections of testicular biopsies from NOA patients exhibited notable vacuolization of some LCs, as well as macrophages stuffed with phagocytized material [216]. LCs may come into direct contact with testicular macrophages or lay near them. This demonstrates a complicated cellular interaction that may be one of the reasons for interstitial tissue changes in NOA [235]. The ultrastructure of LCs in patients with NOA changes significantly. Cells with the unaffected morphology can be detected in the same biopsies, although they are less in number. Because of the increased prevalence of heterochromatin in aberrant LCs, the nucleus was frequently indented. The cell’s cytoplasm contains a large number of low electron density lipid droplets and vacuoles, as well as a decrease in the smooth endoplasmic reticulum cisternae, glycogen, and mitochondria [236]. Histological examination indicates Leydig’s cells hyperplasia (LCH) characterized by a rise in the number of LCs with enlarged nucleoli and reduced lipofuscin. Hyperplastic LCs infiltrate between the seminal tubules and concentrate into multifocal nodules [237].

**Figure 4:**
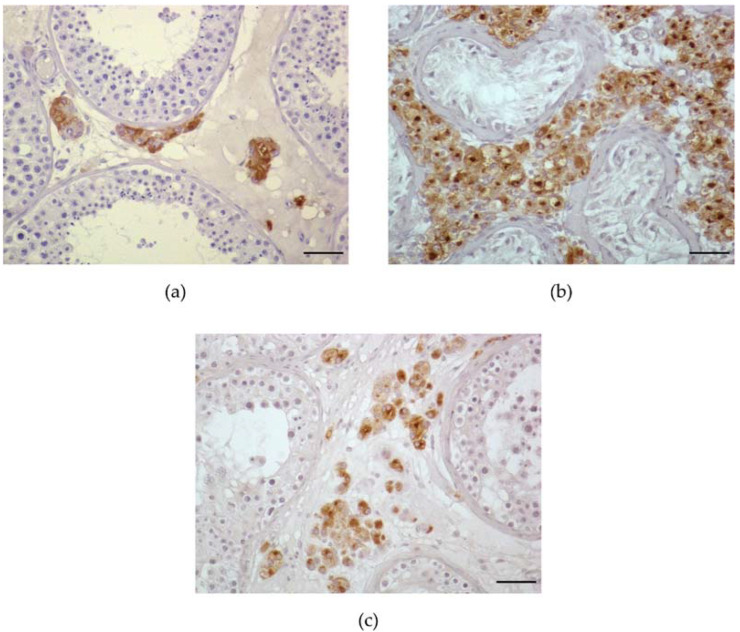
Immunohistochemical staining against insulin-like factor 3 in the mature Leydig cells in testicular biopsies from azoospermic men with different histological patterns. Insulin-like factor 3 expression at protein level is visible as a brown color in Leydig cell cytoplasm (used chromogen: diaminobenzidine—DAB); (**a**) complete spermatogenesis with normal Leydig cells number; (**b**) Sertoli cell only syndrome with exceedingly large Leydig cell hyperplasia; (**c**) mixed atrophy and increased Leydig cell number in the intertubular space. Magnification ×200; scale bar, 50 µm.

The changes in the LCs in NOA patients has been studied for many years. Some studies mention cell hyperplasia [13,195,238,239,240,241,242], while others excluded it [8,234,243,244,245,246]. Even though the proportion of testicular tissue occupied by LCs seems to be larger in histology samples from testes with dysgenetic features, when testis size is taken into account, there is often no significant increase in total Leydig cell volume [8]. Whether or not LCs are truly hyperplastic in men with NOA is still up for debate. In NOA men, LCs often form micronodules, which have been identified as a histological sign of spermatogenic failure. LC micronodules are defined as more than 15 LCs in a cluster [8,13,14]. This is a common finding in those with impaired spermatogenesis (Figure 4) [14]. The presence of large LC clusters has been linked to reproductive disorders such as Klinefelter syndrome [11]. In the testes of rats, phthalate-driven FLC aggregation caused by DBP was previously characterized as Leydig cell tumors [193,247]. Nevertheless, FLC aggregation is not a Leydig cell tumor since FLC quantity is unaffected by DBP exposure [248]. Findings in animals exposed to DBP in utero indicate that inappropriate aggregation of FLCs occurs most likely due to their irregular migration [248]. Another theory is that LC hyperstimulation occurs as a result of increased LH levels compensating for decreasing T levels, resulting in the creation of dysfunctional LC clusters, despite their large size [14]. Furthermore, histological heterogeneity was discovered in LCs coupled into micronodules, implying the presence of cells at various phases of development [53].

Gene set enrichment and protein expression analysis found that the maturation of SCs and LCs was impaired in patients with Klinefelter syndrome. These findings indicate that the cells are immature in adult males with Klinefelter syndrome, and that testicular function impairment begins early in development [249], as indicated by the discovery of the presence of spindle-shaped LCs in biopsies of men with NOA [234]. When compared to normal adult testis biopsy samples, the LC populations in testes with features of dysgenesis have a higher number of undifferentiated cells, as evidenced by increased delta-like homolog 1 (DLK1) and decreased INSL3 expression [53]. ILCs have fewer Reinke crystals (RCs) than mature ALCs, if any. This is consistent with animal studies that indicate immaturity in LCH [248,250].

### 4.3. Endocrine Profile

NOA is a substantial risk factor for androgen insufficiency [251,252,253,254,255,256]. In patients with NOA, serum T levels are commonly found to be low-normal or low [251,253,257]. Low T levels, decreased urogenital masculinization, higher risk of hypospadias and cryptorchidism, and reduced sperm production may all arise from insufficient androgen exposure during MPW [258,259]. The human testis generally generates very moderate amounts of Δ4-C21 steroids, as these are products of the Δ4 pathway: the Δ5 pathway is used throughout androgen production in healthy human testis. In testes with impaired spermatogenesis, however, the Δ4-C21 steroid progesterone builds up in the thickened lamina propria of the seminiferous tubules. Immunohistochemistry indicates that low JS testes express more HSD than high JS testes. Yet, there was no difference in CYP17A1 expression levels across groups. Infertile testes display raised relative HSD levels in their LCs and adopt the Δ4 pathway to convert pregnenolone to T, instead of the Δ5 pathway. This metabolic pathway disruption might be caused by a change in LC density, since in the group with low JS, increased LCs density could enhance the expression of the HSD enzyme [260].

Low T levels are linked to high gonadotropin (LH, FSH) levels, leading to spermatogenesis problems [261,262,263,264,265,266,267]. Several researchers have linked spermatogenesis failure, such as SCOS, MIX, or testicular cancer, to LC dysfunction, which is marked by low T and elevated LH [8,9,10,13,268,269]. A deficiency of T in the body can interfere with spermatogenesis, resulting in spermatogenic cell loss and infertility. The number of SCs in adult men’s testes is the most important factor of sperm production efficiency. The results of a study using mice lacking FSH, FSH receptors, or AR, reveal that both FSH and androgen are necessary for the formation of the full complement of SCs in adult males, while only androgens are essential during fetal and neonatal proliferation, and are required for specific transcript expression during prepubertal development [270]. Thus, reduced T release by FLCs might lead to the suppression of SC proliferation and, as a result, a decrease in the sperm output in adulthood. In some studies, a so-called compensated LC dysfunction was observed, characterized by elevated serum LH levels along with normal levels of total T [9,13,43,240,268,271,272].

It has been suggested that an increase in serum LH levels leads to LCH. Continuous LH stimulation of the LCs can lead to the destruction of the LCs. This overstimulation can result in the production of huge vacuoles and extensive cisterns of the smooth endoplasmic reticulum in LCs. Overstimulated LCs, as well as those with preserved normal form and function, produce enough T to maintain normal blood levels. Furthermore, overstimulated LCs may produce an excess of T, which might explain why a small number of NOA patients had elevated T levels and T overexpression as determined by immunohistochemistry [216]. Since serum T levels may differ between NOA patients, a better indicator of LC function is T/LH ratio. Lardone and colleagues studied factors of LC function (T/LH ratio, Leydig cells/cluster size) along with measures of spermatogenic damage (FSH and testicular volume) in various histological patterns among patients [13]. The T/LH ratio was discovered to be substantially correlated with the number of LCs in a cluster, FSH levels, and testicular volume. These correlations were found to be comparable with the pattern of spermatogenic lesions. In addition, the number of LCs was positively associated with FSH levels and negatively related to testicular volume [13]. Hormonal imbalance has also been connected to the development of LCH and the severity of spermatogenic damage.

In tissues with severe spermatogenesis failure, smaller testicular volume and larger LCs clusters were observed [273]. This can lead to high testicular steroid concentrations per unit volume, but not enough to compensate for serum T levels, as compared to those with normal testicular volumes. As a result, each LC specific hormonal action appears to be altered [13]. In line with prior studies in cancer patients, it was found that the appearance of an expanded LC compartment and LCH in the biopsy contralateral to the tumor-bearing testicle was associated with long-term biochemical signs of LC dysfunction, reflected by decreased serum total T levels and lower total T/LH ratio [274].

Men with NOA have been found to demonstrate high-normal or high levels of E2, as well as a low T to E2 ratio, indicating enhanced aromatase activity [9,273,275,276]. Aromatase P450 (CYP19A1), which is encoded by CYP19, is responsible for the aromatization of androgens into estrogens in various tissues, including the gonads, brain, and adipose tissue. In human testicles, CYP19A1 was localized in LCs, SCs, and germ cells [273]. However, it is the LCs that are the primary site where T is converted to E2 during male adulthood.

**Figure 5:**
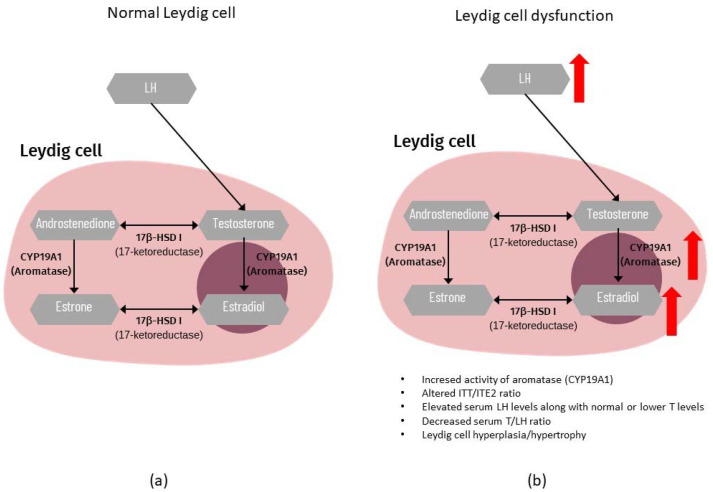
Estrogen synthesis in testes. (**a**) In adulthood, the main site of testicular estrogen production is the Leydig cell. Under the LH stimulation, testosterone (T) and androstendione are converted by aromatase (CYP19A1) to estrone and estradiol (E2), respectively. The proper balance between intratesticular T level (ITT) and estradiol level (ITE2) is crucial for normal testicular function (steroidogenesis and spermatogenesis); (**b**) In testes with impaired spermatogenesis, the dysfunction of Leydig cells is observed. Leydig cell dysfunction is accompanied by increased serum LH levels and decreased T/LH ratio. It was reported that in dysfunctional Leydig cells, the increased activity of aromatase may be observed, which results in elevation of intratesticular E2 levels and thus altered ITT/ITE2 ratio. The increased ITE2 level may be associated with Leydig cell hyperplasia/hypertrophy.

Testicular CYP19A1 appears to be normal in the majority of men with poor spermatogenesis. However, immunohistochemical labeling revealed that men with NOA have higher levels of CYP19A1 transcript and protein expression, and that this was dependent on the severity of spermatogenic dysfunction. No relationship between serum T to E2 ratio or intratesticular testosterone (ITT) to intratesticular estradiol (ITE2) ratio was found, although a link was found between lower ITT and increased ITE2, CYP19A1 expression, and sperm retrieval [277]. The link between CYP19A1 expression in LCs and the ITT/ITE2, but not the serum T/E2, indicates that local aromatase expression altered the intratesticular hormonal milieu [277]. In a certain subset of men with complete SCOS, an altered ITT/ITE2 ratio indicated an increased quantity of CYP19A1, which could lead to LC failure [273]. Additionally, in animal studies, increased ITE2 levels have been associated to LCH and/or LCs hypertrophy [238,278]. This was also confirmed in humans in a study where SCOS patients with low T/LH ratio and LCH had overexpression of CYP19A1 in LCs, which resulted in higher ITE2 levels [279]. Figure 5 presents a schematic illustration of estrogen synthesis in testes.

A recent study examining the quantity of estrogen sulfotransferase and steroid sulfatase mRNA in testicular tissue from SCOS patients found that an unbalanced steroid sulfatase/estrogen sulfotransferase pathway contributes to the testicular hyperestrogenic milieu in individuals with spermatogenic failure and LC dysfunction [280]. Moreover, decreased androgen production in SCOS was associated with lower CYP17A1 expression, which is affected by E2. These findings show that elevated E2 levels in some SCOS patients may play a role in the post-transcriptional degradation of steroidogenic activity in LCs.

Finally, LCs from men with SCOS and symptoms of testicular steroidogenic dysfunction demonstrated a mismatch in the transcriptional and protein expression of CYP17A1, which is associated with lower T synthesis and increased ITE2 [281]. Apart from the clear effects of CYP17A1 mutations and total CYP17A1 deficiency in men with missing or partial external genitalia development [77], milder variants of testicular CYP17A1 deficiency have been associated to idiopathic infertility and hypospadias [282]. Disturbances in male reproductive tract development are caused by a disruption in the androgen–estrogen balance rather than by the action of estrogens alone [283]. Major developmental abnormalities of the male reproductive system related to exposure to potent estrogens can only occur when androgen activity is inhibited at the same time. The latter is brought on by T deficiency [57], LC development complications [57] and, most importantly, the decrease of AR expression [284,285]. It has also been revealed that the progesterone receptor (PR) and ER have a role in the pathophysiology of the MA and SCOS phenotypes in infertile males. In MA and SCOS patients, PR expression was decreased in all cell types when compared to OA patients, with only the truncated variant of PR present in SCOS. ER expression was decreased in the spermatogenic cells and LCs of MA testes, but enhanced in the LCs of SCOS testes [286]. Mizuno et al. [287] conducted a study on cryptorchid rats with impaired spermatogenesis. They hypothesized that higher ESR1expression in the LCs of cryptorchid testes is related to E2 levels in the testicular tissue, and that an androgen–estrogen imbalance impairs spermatogenesis in cryptorchidism.

Along with T and E2, INSL3 is a crucial secretory product of testicular LCs. Serum INSL3 reflects LC maturity and function [288]. Inhibiting the HPG axis alters INSL3 production [44]. INSL3 levels in the blood can be reduced in people with hypogonadism, such as those with Klinefelter syndrome [43,289,290] and Kallmann syndrome [291]. Mutations in the INSL3 or RXFP2 genes cause improper testicular descent during embryogenesis in rats, and it is a rare cause of cryptorchidism in humans [42,292,293,294]. INSL3 circulating concentration has mostly been evaluated as a possible marker for LC activity and as a potential prognostic tool for developmental and reproductive disorders in men [295]. T and E2 may influence INSL3 gene transcription by attaching to their receptors, activating or inhibiting transcription factors SF1 and NUR77, respectively [295,296]. The INSL3 promoter contains a testosterone-responsive element that acts as a binding site for NUR77 and SF1 [297]; several hypotheses about the role of estrogens have been proposed, including estradiol-mediated disruption of NUR77 and SF1 acetylation status and antagonism between AR and ER, but the exact mechanisms remain unknown [298].

### 4.4. Testicular Dysgenesis Syndrome

Growing epidemiological evidence points to a direct cause-and-effect relationship between prenatal estrogen exposure and various pathologies of the male reproductive tract, such as hypospadias, cryptorchidism, abnormal spermatogenesis, and testicular cancer, which have been grouped together under the name *testicular dysgenesis syndrome* (TDS) [16]. Testicular dysgenesis during fetal development can result in a primary failure of spermatogenesis and impairment of LCs. Micronodules are a common finding in those with impaired spermatogenesis and other TDS-related disorders [15]. In normally descending TDS testes, LCs within micronodules demonstrate a lack of RCs, which may be a characteristic of recently renewed ILCs [299]. Altered serum levels of reproductive hormones and reduced size and volume of male reproductive organs are other characteristics, which may also be part of the syndrome [300,301]. Although TDS and NOA share many common characteristics, not every case of TDS will be associated with simultaneous occurrence of NOA and vice versa. Men with TDS may only have slightly reduced semen quality and be fertile or subfertile [300]. According to the TDS theory, genetic and/or environmental factors cause a failure in SC and LC differentiation as a fundamental change in early fetal development. As a result, germ cell proliferation and T production are impeded [16,302]. TDS is also associated with altered estrogen levels. Cryptorchidism, epididymal abnormalities, infertility, and testicular cancer have all been linked to estrogen and xenoestrogen exposure during fetal and neonatal development [303]. A growing body of epidemiological evidence suggests a direct causal link between prenatal estrogen exposure and a variety of male reproductive system disorders grouped together as TDS [16]. Moreover, TDS is also associated with a mutation of INSL3 [45].

## 5. Conclusions

In summary, infertile men with spermatogenic failure exhibit substantial evidence of LC dysfunction. The association between impaired spermatogenesis and altered LCs activity might be related to an aberrant paracrine connection between the seminiferous epithelium and LCs. On the other hand, impairment of spermatogenesis and LC function may result from congenital insufficiency of both compartments arising during abnormal fetal/infant development. LCs micronodules are prevalent in human testicular biopsies, specifically in regions with severe spermatogenic insufficiency and an imbalance in the hormonal ratios, promoting the paracrine connection hypothesis between germ cells and LCs. Animal models were designed to investigate the mechanisms underlying diseases associated with prenatal-initiated spermatogenesis failure, as these events are difficult to analyze in humans. These trials provided compelling evidence of a link between somatic cell dysfunction and diseases associated with NOA and/or TDS. However, the mechanism by which defects in fetal testicular development cause reproductive difficulties in adult males, such as infertility, still remains unknown. Much research has been done on the origins and development of the LCs since they were originally reported in 1850 by Franz Leydig. Still, little is known about the ontogenesis of LCs and the relationship between FLCs and ALCs. Determining how LCs are formed will undoubtedly help to understand the development of the male reproductive system, as well as the mechanisms underlying TDS and T deficiency, and how environmental factors can affect the male reproductive system.

## Figures and Tables

**Table 1:** Direct causes underlying non-obstructive azoospermia.

**Etiology**	**Example**	**References**
Chromosomal	Klinefelter syndrome	[11,149,151]
	Y-chromosome microdeletions	[152,153,154,155]
Genetic	Autosomal monogenic factors (TEX11, NR5A1, SYCP3, MEI1, and others)	[156,157,158,159,160,161,162,163,164,165]
Hormonal	Kallmann syndrome	[166]
	Acquired hypogonadotropic hypogonadism	[167,168,169]
	Hyperprolactinemia	[170,171]
	Androgen resistance	[172,173]
Developmental/Structural	Cryptorchidism	[174,175,176]
	Varicocele	[177,178,179,180,181,182]
Radiation and toxins	Radiotherapy	[183,184,185]
	Chemotherapy	[184,186,187]
	Drugs	[188,189,190,191,192]
	EDCs (phthalates, bisphenol A)	[193,194,195,196,197,198,199,200]
	Alcohol abuse	[201,202]
Infections	Mumps orchitis	[203,204]
	Others	[205,206,207]
Testicular trauma	Torsion	[208,209]
Other exogenous factors	Heat	[210,211]
Idiopathic	-	[212,213,214]

## Data Availability

Not applicable.

## References

[1] Lara N.L.M., Costa G.M.J., Avelar G.F., Lacerda S.M.S.N., Hess R.A., de França L.R., Skinner M. (2018). Testis physiology—Overview and histology. Encyclopedia of Reproduction.

[2] Svingen T., Koopman P. (2013). Building the mammalian testis: Origins, differentiation, and assembly of the component cell populations. Genes Dev..

[3] Ross M.H., Pawlina W. (2015). Male reproductive system. Histology: A Text and Atlas.

[4] Fietz D., Bergmann M., Simoni M., Huhtaniemi I.T. (2017). Functional Anatomy and Histology of the Testis. Endocrinology of the Testis and Male Reproduction.

[5] Jones R.E., Lopez K.H. (2014). The male reproductive system. Human Reproductive Biology.

[6] O’Shaughnessy P.J., Winters S., Huhtaniem I.T. (2017). The human Leydig cell. Male Hypogonadism.

[7] Wallace E.M., Groome N.P., Riley S.C., Parker A.C., Wu F.C.W. (1997). Effects of chemotherapy-induced testicular damage on inhibin, gonadotropin, and testosterone secretion: A prospective longitudinal study. J. Clin. Endocrinol. Metab..

[8] Holm M., De Meyts E.R., Andersson A.M., Skakkebæk N.E. (2003). Leydig cell micronodules are a common finding in testicular biopsies from men with impaired spermatogenesis and are associated with decreased testosterone/LH ratio. J. Pathol..

[9] Andersson A.-M., Jørgensen N., Frydelund-Larsen L., Meyts E.R.-D., Skakkebæk N.E. (2004). Impaired Leydig Cell Function in Infertile Men: A Study of 357 Idiopathic Infertile Men and 318 Proven Fertile Controls. J. Clin. Endocrinol. Metab..

[10] De Kretser D.M. (2004). Is Spermatogenic Damage Associated with Leydig Cell Dysfunction?. J. Clin. Endocrinol. Metab..

[11] Wikström A.M., Dunkel L. (2011). Klinefelter syndrome. Best Pract. Res. Clin. Endocrinol. Metab..

[12] Adamczewska D., Slowikowska-Hilczer J., Marchlewska K., Walczak-Jedrzejowska R. (2020). Features of gonadal dysgenesis and Leydig cell impairment in testes with Sertoli cell-only syndrome. Folia Histochem. Cytobiol..

[13] Lardone M.C., Piottante A., Valdevenito R., Ebensperger M., Castro A. (2012). Histological and hormonal testicular function in oligo/azoospermic infertile men. Andrologia.

[14] Joensen U.N., Jørgensen N., Meyts E.R., De Skakkebæk N.E. (2008). Testicular dysgenesis syndrome and Leydig cell function. Basic Clin. Pharmacol. Toxicol..

[15] Sharpe R.M. (2020). Androgens and the masculinization programming window: Human–rodent differences. Biochem. Soc. Trans..

[16] Skakkebæk N., Meyts E.R.-D., Main K.M. (2001). Testicular dysgenesis syndrome: An increasingly common developmental disorder with environmental aspects: Opinion. Hum. Reprod..

[17] Zirkin B.R., Papadopoulos V. (2018). Leydig cells: Formation, function, and regulation. Biol. Reprod..

[18] Griswold S., Behringer R. (2009). Fetal Leydig cell origin and development. Sex. Dev..

[19] Teerds K.J., Huhtaniemi I.T. (2015). Morphological and functional maturation of Leydig cells: From rodent models to primates. Hum. Reprod. Updat..

[20] Chen H., Wang Y., Ge R., Zirkin B.R. (2017). Leydig cell stem cells: Identification, proliferation and differentiation. Mol. Cell. Endocrinol..

[21] Nistal M., Paniagua R., Regadera J., Santamarìa L., Amat P. (1986). A quantitative morphological study of human Leydig cells from birth to adulthood. Cell Tissue Res..

[22] Prince F.P. (1990). Ultrastructural evidence of mature Leydig cells and Leydig cell regression in the neonatal human testis. Anat. Rec..

[23] Prince F.P. (1992). Ultrastructural evidence of indirect and direct autonomic innervation of human Leydig cells: Comparison of neonatal, childhood and pubertal ages. Cell Tissue Res..

[24] Prince F.P. (2001). The triphasic nature of Leydig cell development in humans, and comments on nomenclature. J. Endocrinol..

[25] Habert R., Lejeune H., Saez J.M. (2001). Origin, differentiation and regulation of fetal and adult Leydig cells. Mol. Cell. Endocrinol..

[26] Yao H.H.-C., Whoriskey W., Capel B. (2002). Desert Hedgehog/Patched 1 signaling specifies fetal Leydig cell fate in testis organogenesis. Genes Dev..

[27] Miyabayashi K., Katoh-Fukui Y., Ogawa H., Baba T., Shima Y., Sugiyama N., Kitamura K., Morohashi K.-I. (2013). Aristaless Related Homeobox Gene, Arx, Is Implicated in Mouse Fetal Leydig Cell Differentiation Possibly through Expressing in the Progenitor Cells. PLoS ONE.

[28] Codesal J., Regadera J., Nistal M., Regadera-Sejas J., Paniagua R. (1990). Involution of human fetal Leydig cells. An immunohistochemical, ultrastructural and quantitative study. J. Anat..

[29] Gautier C., Levacher C., Saez J.M., Habert R. (1997). Expression and regulation of transforming growth factor beta1 mRNA and protein in rat fetal testis in vitro. Biochem. Biophys. Res. Commun..

[30] Rouiller-Fabre V., Carmona S., Merhi R.A., Cate R., Habert R., Vigier B. (1998). Effect of Anti-Mullerian Hormone on Sertoli and Leydig Cell Functions in Fetal and Immature Rats. Endocrinology.

[31] Svechnikov K., Landreh L., Weisser J., Izzo G., Colón E., Svechnikova I., Söder O. (2010). Origin, Development and Regulation of Human Leydig Cells. Horm. Res. Paediatr..

[32] Kuopio T., Paranko J., Pelliniemi L.J. (1989). Basement membrane and epithelial features of fetal-type Leydig cells in rat and human testis. Differentiation.

[33] O’Shaughnessy P.J., Baker P.J., Heikkilä M., Vainio S., Mcmahon A.P. (2000). Localization of 17beta-hydroxysteroid dehydrogenase/17-ketosteroid reductase isoform expression in the developing mouse testis--androstenedione is the major androgen secreted by fetal/neonatal leydig cells. Endocrinology.

[34] Shima Y., Miyabayashi K., Haraguchi S., Arakawa T., Otake H., Baba T., Matsuzaki S., Shishido Y., Akiyama H., Tachibana T. (2013). Contribution of Leydig and Sertoli Cells to Testosterone Production in Mouse Fetal Testes. Mol. Endocrinol..

[35] Blaschko S.D., Cunha G.R., Baskin L.S. (2012). Molecular mechanisms of external genitalia development. Differentiation.

[36] Baskin L., Shen J., Sinclair A., Cao M., Liu X., Liu G., Isaacson D., Overland M., Li Y., Cunha G.R. (2018). Development of the human penis and clitoris. Differentiation.

[37] Rao P.K., Burnett A.L., Kavoussi P., Costabile R., Salonia A. (2013). Development of the Male Reproductive System. Clinical Urologic Endocrinology.

[38] Hutson J.M., Hutson J.M., Warne G.L., Grover S.R. (2012). Embryology of the human genital tract. Disorders of Sex Development.

[39] Ge R., Chen G., Tanrikut C., Hardy M. (2007). Phthalate ester toxicity in Leydig cells: Developmental timing and dosage considerations. Reprod. Toxicol..

[40] Kumagai J., Hsu S.Y., Matsumi H., Roh J.S., Fu P., Wade J.D., Bathgate R.D.A., Hsueh A.J.W. (2002). INSL3/Leydig insulin-like peptide activates the LGR8 receptor important in testis descent. J. Biol. Chem..

[41] Bogatcheva N.V., Truong A., Feng S., Engel W., Adham I.M., Agoulnik A.I. (2003). GREAT/LGR8 is the only receptor for insulin-like 3 peptide. Mol. Endocrinol..

[42] Ivell R., Hartung S. (2003). The molecular basis of cryptorchidism. Mol. Hum. Reprod..

[43] Bay K., Hartung S., Ivell R., Schumacher M., Jürgensen D., Jørgensen N., Holm M., Skakkebaek N.E., Andersson A.-M. (2005). Insulin-Like Factor 3 Serum Levels in 135 Normal Men and 85 Men with Testicular Disorders: Relationship to the Luteinizing Hormone-Testosterone Axis. J. Clin. Endocrinol. Metab..

[44] Bay K., Matthiesson K.L., McLachlan R.I., Andersson A.-M. (2006). The Effects of Gonadotropin Suppression and Selective Replacement on Insulin-Like Factor 3 Secretion in Normal Adult Men. J. Clin. Endocrinol. Metab..

[45] Ferlin A., Bogatcheva N., Gianesello L., Pepe A., Vinanzi C., Agoulnik A., Foresta C. (2006). Insulin-like factor 3 gene mutations in testicular dysgenesis syndrome: Clinical and functional characterization. Mol. Hum. Reprod..

[46] Foresta C., Bettella A., Vinanzi C., Dabrilli P., Meriggiola M.C., Garolla A., Ferlin A. (2004). A Novel Circulating Hormone of Testis Origin in Humans. J. Clin. Endocrinol. Metab..

[47] Berensztein E., Belgorosky A., De Dávila M.T.G., Rivarola M.A. (1995). Basal Testosterone Secretion and Response to Human Luteinizing, Follicle-Stimulating, and Growth Hormones in Culture of Cells Isolated from Testes of Infants and Children. Pediatr. Res..

[48] Ye L., Li X., Li L., Chen H., Ge R.-S. (2017). Insights into the Development of the Adult Leydig Cell Lineage from Stem Leydig Cells. Front. Physiol..

[49] MacLeod D.J., Sharpe R.M., Welsh M., Fisken M., Scott H.M., Hutchison G.R., Drake A.J., van den Driesche S. (2010). Androgen action in the masculinization programming window and development of male reproductive organs. Int. J. Androl..

[50] Ge R.-S., Dong Q., Sottas C.M., Chen H., Zirkin B.R., Hardy M.P. (2005). Gene Expression in Rat Leydig Cells During Development from the Progenitor to Adult Stage: A Cluster Analysis. Biol. Reprod..

[51] Ge R.-S., Dong Q., Sottas C.M., Papadopoulos V., Zirkin B.R., Hardy M.P. (2006). In search of rat stem Leydig cells: Identification, isolation, and lineage-specific development. Proc. Natl. Acad. Sci. USA.

[52] Stanley E.L., Johnston D.S., Fan J., Papadopoulos V., Chen H., Ge R.-S., Zirkin B.R., Jelinsky S.A. (2011). Stem Leydig Cell Differentiation: Gene Expression During Development of the Adult Rat Population of Leydig Cells. Biol. Reprod..

[53] Lottrup G., Nielsen J., Maroun L., Møller L., Yassin M., Leffers H., Skakkebæk N., Meyts E.R.-D. (2014). Expression patterns of DLK1 and INSL3 identify stages of Leydig cell differentiation during normal development and in testicular pathologies, including testicular cancer and Klinefelter syndrome. Hum. Reprod..

[54] Ariyaratne S., Kim I., Mills N., Mason I., Mendis-Handagama C. (2003). Effects of ethane dimethane sulfonate on the functional structure of the adult rat testis. Arch. Androl..

[55] Davidoff M.S., Middendorff R., Enikolopov G., Riethmacher D., Holstein A.F., Müller D. (2004). Progenitor cells of the testosterone-producing Leydig cells revealed. J. Cell Biol..

[56] Hardy M.P., Sharma R.S., Arambepola N.K., Sottas C.M., Russell L.D., Bunick D., Hess R., Cooke P.S. (1996). Increased proliferation of Leydig cells induced by neonatal hypothyroidism in the rat. J. Androl..

[57] Sharpe R.M., Rivas A., Walker M., Mckinnell C., Fisher J.S. (2003). Effect of neonatal treatment of rats with potent or weak (environmental) oestrogens, or with a GnRH antagonist, on Leydig cell development and function through puberty into adulthood. Int. J. Androl..

[58] Rajpert-De Meyts E., Almstrup K., Nielsen J.E., Skakkebæk N.E., Ježek D. (2013). The Testis in Childhood between Birth and Puberty. Atlas on the Human Testis.

[59] Haider S.G. (2004). Cell Biology of Leydig Cells in the Testis. Int. Rev. Cytol..

[60] Barsoum I.B., Kaur J., Ge R.S., Cooke P.S., Yao H.H. (2013). Dynamic changes in fetal Leydig cell populations influence adult Leydig cell populations in mice. FASEB J..

[61] Kaftanovskaya E.M., Lopez C., Ferguson L., Myhr C., Agoulnik A.I. (2015). Genetic ablation of androgen receptor signaling in fetal Leydig cell lineage affects Leydig cell functions in adult testis. FASEB J..

[62] Shima Y., Matsuzaki S., Miyabayashi K., Otake H., Baba T., Kato S., Huhtaniemi I., Morohashi K.-I. (2015). Fetal Leydig Cells Persist as an Androgen-Independent Subpopulation in the Postnatal Testis. Mol. Endocrinol..

[63] O’Shaughnessy P., Willerton L., Baker P. (2002). Changes in Leydig Cell Gene Expression during Development in the Mouse. Biol. Reprod..

[64] Chen H., Ge R.-S., Zirkin B.R. (2009). Leydig cells: From stem cells to aging. Mol. Cell. Endocrinol..

[65] Stévant I., Neirijnck Y., Borel C., Escoffier J., Smith L.B., Antonarakis S.E., Dermitzakis E.T., Nef S. (2018). Deciphering Cell Lineage Specification during Male Sex Determination with Single-Cell RNA Sequencing. Cell Rep..

[66] Inoue M., Shima Y., Miyabayashi K., Tokunaga K., Sato T., Baba T., Ohkawa Y., Akiyama H., Suyama M., Morohashi K.-I. (2016). Isolation and Characterization of Fetal Leydig Progenitor Cells of Male Mice. Endocrinology.

[67] Pelosi E., Koopman P. (2017). Development of the Testis. Ref. Modul. Biomed. Sci..

[68] Su D.-M., Feng Y., Wang L., Wu Y.-L., Ge R.-S., Ma X. (2018). Influence of fetal Leydig cells on the development of adult Leydig cell population in rats. J. Reprod. Dev..

[69] Kilcoyne K.R., Smith L.B., Atanassova N., Macpherson S., McKinnell C., van den Driesche S., Jobling M.S., Chambers T.J.G., De Gendt K., Verhoeven G. (2014). Fetal programming of adult Leydig cell function by androgenic effects on stem/progenitor cells. Proc. Natl. Acad. Sci. USA.

[70] Shima Y., Morohashi K.-i. (2017). Leydig progenitor cells in fetal testis. Mol. Cell Endocrinol..

[71] Heinrich A., DeFalco T. (2020). Essential roles of interstitial cells in testicular development and function. Andrology.

[72] Bott R.C., McFee R.M., Clopton D.T., Toombs C., Cupp A.S. (2006). Vascular Endothelial Growth Factor and Kinase Domain Region Receptor Are Involved in Both Seminiferous Cord Formation and Vascular Development During Testis Morphogenesis in the Rat. Biol. Reprod..

[73] Combes A.N., Wilhelm D., Davidson T., Dejana E., Harley V., Sinclair A., Koopman P. (2009). Endothelial cell migration directs testis cord formation. Dev. Biol..

[74] DeFalco T., Bhattacharya I., Williams A.V., Sams D.M., Capel B. (2014). Yolk-sac–derived macrophages regulate fetal testis vascularization and morphogenesis. Proc. Natl. Acad. Sci. USA.

[75] Potter S.J., DeFalco T. (2017). Role of the testis interstitial compartment in spermatogonial stem cell function. Reproduction.

[76] Zhou R., Wu J., Liu B., Jiang Y., Chen W., Li J., He Q., He Z. (2019). The roles and mechanisms of Leydig cells and myoid cells in regulating spermatogenesis. Cell Mol. Life Scixp..

[77] Miller W.L., Auchus R.J. (2011). The Molecular Biology, Biochemistry, and Physiology of Human Steroidogenesis and Its Disorders. Endocr. Rev..

[78] Stocco D.M. (2001). StAR Protein and the Regulation of Steroid Hormone Biosynthesis. Annu. Rev. Physiol..

[79] Hauet T., Liu J., Li H., Gazouli M., Culty M., Papadopoulos V. (2002). PBR, Star, and PKA: Partners in cholesterol transport in steroidogenic cells. Endocr. Res..

[80] Payne A.H., Hales D.B. (2004). Overview of Steroidogenic Enzymes in the Pathway from Cholesterol to Active Steroid Hormones. Endocr. Rev..

[81] Scott H.M., Mason J.I., Sharpe R.M. (2009). Steroidogenesis in the Fetal Testis and Its Susceptibility to Disruption by Exogenous Compounds. Endocr. Rev..

[82] Aghazadeh Y., Zirkin B.R., Papadopoulos V. (2015). Pharmacological Regulation of the Cholesterol Transport Machinery in Steroidogenic Cells of the Testis. Vitam. Horm..

[83] Schiffer L., Arlt W., Storbeck K.-H. (2018). Intracrine androgen biosynthesis, metabolism and action revisited. Mol. Cell. Endocrinol..

[84] Walker W.H. (2009). Molecular mechanisms of testosterone action in spermatogenesis. Steroids.

[85] Connan-Perrot S., Léger T., Lelandais P., Desdoits-Lethimonier C., David A., Fowler P., Mazaud-Guittot S. (2021). Six Decades of Research on Human Fetal Gonadal Steroids. Int. J. Mol. Sci..

[86] Walker W.H. (2011). Testosterone signaling and the regulation of spermatogenesis. Spermatogenesis.

[87] De Gent K., Swinnen J.K., Saunders P.T.K., Schoonjas L., Dewerchin M., Devoss A., Tan K., Atanassova N., Claessens F., Lecureuil C. (2004). Faculty Opinions recommendation of A Sertoli cell-selective knockout of the androgen receptor causes spermatogenic arrest in meiosis. Proc. Natl. Acad. Sci. USA.

[88] Welsh M., Saunders P., Atanassova N., Sharpe R., Smith L.B. (2009). Androgen action via testicular peritubular myoid cells is essential for male fertility. FASEB J..

[89] O’Shaughnessy P.J., Verhoeven G., De Gendt K., Monteiro A., Abel M.H. (2010). Direct Action through the Sertoli Cells Is Essential for Androgen Stimulation of Spermatogenesis. Endocrinology.

[90] Willems A., Roesl C., Mitchell R., Milne L., Jeffery N., Smith S., Verhoeven G., Brown P., Smith L. (2015). Sertoli cell androgen receptor signalling in adulthood is essential for post-meiotic germ cell development. Mol. Reprod. Dev..

[91] Tsai M.-Y., Yeh S.-D., Wang R.-S., Yeh S., Zhang C., Lin H.-Y., Tzeng C.-R., Chang C. (2006). Differential effects of spermatogenesis and fertility in mice lacking androgen receptor in individual testis cells. Proc. Natl. Acad. Sci. USA.

[92] Zhou Q., Nie R., Prins G.S., Saunders P., Katzenellenbogen B.S., Hess R.A. (2002). Localization of androgen and estrogen receptors in adult male mouse reproductive tract. J. Androl..

[93] Smith L.B., Walker W.H. (2014). The regulation of spermatogenesis by androgens. Semin. Cell Dev. Biol..

[94] Willems A., Batlouni S.R., Esnal A., Swinnen J.V., Saunders P.T.K., Sharpe R.M., França L.R., De Gendt K., Verhoeven G. (2010). Selective Ablation of the Androgen Receptor in Mouse Sertoli Cells Affects Sertoli Cell Maturation, Barrier Formation and Cytoskeletal Development. PLoS ONE.

[95] Meng J., Greenlee A.R., Taub C.J., Braun R.E. (2011). Sertoli Cell-Specific Deletion of the Androgen Receptor Compromises Testicular Immune Privilege in Mice. Biol. Reprod..

[96] Chang C., Chen Y.-T., Yeh S.-D., Xu Q., Wang R.-S., Guillou F., Lardy H., Yeh S. (2004). Infertility with defective spermatogenesis and hypotestosteronemia in male mice lacking the androgen receptor in Sertoli cells. Proc. Natl. Acad. Sci. USA.

[97] Holdcraft R.W., Braun R.E. (2004). Androgen receptor function is required in Sertoli cells for the terminal differentiation of haploid spermatids. Development.

[98] Wang R.-S., Yeh S., Chen L.-M., Lin H.-Y., Zhang C., Ni J., Wu C.-C., di Sant’Agnese P.A., Demesy-Bentley K.L., Tzeng C.-R. (2006). Androgen Receptor in Sertoli Cell Is Essential for Germ Cell Nursery and Junctional Complex Formation in Mouse Testes. Endocrinology.

[99] Mayer C., Ádám M., Walenta L., Schmid N., Heikela H., Schubert K., Flenkenthaler F., Dietrich K.-G., Gruschka S., Arnold G.J. (2018). Insights into the role of androgen receptor in human testicular peritubular cells. Andrology.

[100] Carreau S., Wolczynski S., Galeraud-Denis I. (2010). Aromatase, oestrogens and human male reproduction. Philos. Trans. R. Soc. B Biol. Sci..

[101] Fietz D., Ratzenböck C., Hartmann K., Raabe O., Kliesch S., Weidner W., Klug J., Bergmann M. (2014). Expression pattern of estrogen receptors α and β and G-protein-coupled estrogen receptor 1 in the human testis. Histochem. Cell Biol..

[102] Oliveira P.F., Alves M.G., Martins A.D., Correia S., Bernardino R.L., Silva J., Barros A., Sousa M., Cavaco J.E., Socorro S. (2014). Expression pattern of G protein-coupled receptor 30 in human seminiferous tubular cells. Gen. Comp. Endocrinol..

[103] Bernardino R., Alves M.G., Silva J., Barros A., Ferraz L., Sousa M., Sa R., Oliveira P.F. (2016). Expression of Estrogen Receptors Alpha (ER-α), Beta (ER-β), and G Protein-Coupled Receptor 30 (GPR30) in Testicular Tissue of Men with Klinefelter Syndrome. Horm. Metab. Res..

[104] Abney T.O. (1999). The potential roles of estrogens in regulating Leydig cell development and function: A review. Steroids.

[105] Chen B., Chen D., Jiang Z., Li J., Liu S., Dong Y., Yao W., Akingbemi B., Ge R., Li X. (2014). Effects of Estradiol and Methoxychlor on Leydig Cell Regeneration in the Adult Rat Testis. Int. J. Mol. Sci..

[106] Vaucher L., Funaro M.G., Mehta A., Mielnik A., Bolyakov A., Prossnitz E.R., Schlegel P.N., Paduch D. (2014). Activation of GPER-1 Estradiol Receptor Downregulates Production of Testosterone in Isolated Rat Leydig Cells and Adult Human Testis. PLoS ONE.

[107] Pentikäinen V., Erkkilä K., Suomalainen L., Parvinen M., Dunkel L. (2000). Estradiol Acts as a Germ Cell Survival Factor in the Human Testis in Vitro. J. Clin. Endocrinol. Metab..

[108] Robertson K.M., O’Donnell L., Simpson E.R., Jones M.E.E. (2002). The Phenotype of the Aromatase Knockout Mouse Reveals Dietary Phytoestrogens Impact Significantly on Testis Function. Endocrinology.

[109] Walczak-Jędrzejowska R., Slowikowska-Hilczer J., Marchlewska K., Kula K. (2008). Maturation, proliferation and apoptosis of seminal tubule cells at puberty after administration of estradiol, follicle stimulating hormone or both. As. J. Androl..

[110] Chimento A., Sirianni R., Delalande C., Silandre D., Bois C., Andò S., Maggiolini M., Carreau S., Pezzi V. (2010). 17 beta-estradiol activates rapid signaling pathways involved in rat pachytene spermatocytes apoptosis through GPR30 and ER alpha. Mol. Cell Endocrinol..

[111] Royer C., Lucas T.F.G., Lazari M.F.M., Porto C.S. (2012). 17Beta-Estradiol Signaling and Regulation of Proliferation and Apoptosis of Rat Sertoli Cells. Biol. Reprod..

[112] Chimento A., Sirianni R., Casaburi I., Pezzi V. (2014). Role of estrogen receptors and G protein-coupled estrogen receptor in regulation of hypothalamus-pituitary-testis axis and spermatogenesis. Front Endocrinol. (Lausanne).

[113] Guarducci E., Nuti F., Becherini L., Rotondi M., Balercia G., Forti G., Krausz C. (2006). Estrogen receptor α promoter polymorphism: Stronger estrogen action is coupled with lower sperm count. Hum. Reprod..

[114] Cho H.W., Nie R., Carnes K., Zhou Q., Sharief N.A.Q., Hess R.A. (2003). The antiestrogen ICI 182,780 induces early effects on the adult male mouse reproductive tract and long-term decreased fertility without testicular atrophy. Reprod. Biol. Endocrinol..

[115] Robertson K.M., O’Donnell L., Jones M.E.E., Meachem S.J., Boon W.C., Fisher C.R., Graves K.H., McLachlan R.I., Simpson E.R. (1999). Impairment of spermatogenesis in mice lacking a functional aromatase (*cyp 19*) gene. Proc. Natl. Acad. Sci. USA.

[116] Griffeth R.J., Bianda V., Nef S. (2014). The emerging role of insulin-like growth factors in testis development and function. Basic Clin. Androl..

[117] Cannarella R., Condorelli R.A., La Vignera S., Calogero A.E. (2018). Effects of the insulin-like growth factor system on testicular differentiation and function: A review of the literature. Andrology.

[118] Guo C., Cho K.-S., Li Y., Tchedre K., Antolik C., Ma J., Chew J., Utheim T.P., Huang X.A., Yu H. (2018). IGFBPL1 Regulates Axon Growth through IGF-1-mediated Signaling Cascades. Sci. Rep..

[119] Hermann B.P., Cheng K., Singh A., Roa-De La Cruz L., Mutoji K.N., Chen I.-C., Gildersleeve H., Lehle J.D., Mayo M., Westernströer B. (2018). The Mammalian Spermatogenesis Single-Cell Transcriptome, from Spermatogonial Stem Cells to Spermatids. Cell Rep..

[120] Neirijnck Y., Papaioannou M.D., Nef S. (2019). The Insulin/IGF System in Mammalian Sexual Development and Reproduction. Int. J. Mol. Sci..

[121] Neirijnck Y., Calvel P., Kilcoyne K.R., Kühne F., Stévant I., Griffeth R.J., Pitetti J.-L., Andric S.A., Hu M.-C., Pralong F. (2018). Insulin and IGF1 receptors are essential for the development and steroidogenic function of adult Leydig cells. FASEB J..

[122] Thackare H., Nicholson H.D., Whittington K. (2006). Oxytocin—Its role in male reproduction and new potential therapeutic uses. Hum. Reprod. Updat..

[123] Assinder S.J., Rezvani A., Nicholson H.D. (2002). Oxytocin promotes spermiation and sperm transfer in the mouse. Int. J. Androl..

[124] Kawamura K., Kumagai J., Sudo S., Chun S.-Y., Pisarska M., Morita H., Toppari J., Fu P., Wade J.D., Bathgate R.A.D. (2004). Paracrine regulation of mammalian oocyte maturation and male germ cell survival. Proc. Natl. Acad. Sci. USA.

[125] Amory J.K., Page S.T., Anawalt B.D., Coviello A.D., Matsumoto A.M., Bremner W.J. (2007). Elevated End-of-Treatment Serum INSL3 Is Associated with Failure to Completely Suppress Spermatogenesis in Men Receiving Male Hormonal Contraception. J. Androl..

[126] Francomano D., Sanguigni V., Capogrosso P., Deho F., Antonini G. (2021). New Insight into Molecular and Hormonal Connection in Andrology. Int. J. Mol. Sci..

[127] Meinhardt A., Bacher M., McFarlane J.R., Metz C.N., Seitz J., Hedger M.P., de Kretser D.M., Bucala M. (1996). Macrophage migration inhibitory factor production by Leydig cells: Evidence for a role in the regulation of testicular function. Endocrinology.

[128] Wennemuth G., Aumüller G., Bacher M., Meinhardt A. (2000). Macrophage Migration Inhibitory Factor-Induced Ca^2+^ Response in Rat Testicular Peritubular Cells. Biol. Reprod..

[129] Zhang Y.-Q., He X.-Z., Zhang J.-S., Wang R.-A., Zhou J., Xu R.-J. (2004). Stage-specific localization of transforming growth factor beta1 and beta3 and their receptors during spermatogenesis in men. As. J. Androl..

[130] Gonzalez C.R., Gonzalez B., Rulli S.B., Huhtaniemi I., Calandra R.S., Gonzalez-Calvar S.I. (2010). TGF-.BETA.1 System in Leydig Cells. Part I: Effect of hCG and Progesterone. J. Reprod. Dev..

[131] Gonzalez C.R., Calandra R.S., Gonzalez-Calvar I.S. (2012). Influence of the photoperiod on TGF-β1 and p15 expression in hamster Leydig cells. Reprod. Biol..

[132] Hart P.J., Deep S., Taylor A.B., Shu Z., Hinck C.S., Hinck A.P. (2002). Crystal structure of the human TβR2 ectodomain–TGF-β3 complex. Nat. Struct. Biol..

[133] Ebner R., Chen R.-H., Lawler S., Zioncheck T., Derynck R. (1993). Determination of Type I Receptor Specificity by the Type II Receptors for TGF-β or Activin. Science.

[134] Loveland K.L., Klein B., Pueschl D., Indumathy S., Bergmann M., Loveland B.E., Hedger M.P., Schuppe H.-C. (2017). Cytokines in Male Fertility and Reproductive Pathologies: Immunoregulation and Beyond. Front. Endocrinol..

[135] Eldamnhoury E.M., Elatrash G.A., Rashwan H.M., El-Sakka A.I. (2018). Association between leukocytospermia and semen interleukin-6 and tumor necrosis factor-alpha in infertile men. Andrology.

[136] Boivin J., Bunting L., Collins J.A., Nygren K.G. (2007). International estimates of infertility prevalence and treatment-seeking: Potential need and demand for infertility medical care. Hum. Reprod..

[137] Jungwirth A., Giwercman A., Tournaye H., Diemer T., Kopa Z., Dohle G., Krausz C., EAU Working Group on Male Infertility (2012). European Association of Urology Guidelines on Male Infertility: The 2012 Update. Eur. Urol..

[138] Massart A., Lissens W., Tournaye H., Stouffs K. (2012). Genetic causes of spermatogenic failure. As. J. Androl..

[139] Chiba K., Enatsu N., Fujisawa M. (2016). Management of non-obstructive azoospermia. Reprod. Med. Biol..

[140] Skakkebaek N.E., Rajpert-De Meyts E., Buck Louis G.M., Toppari J., Andersson A.-M., Eisenberg M.L., Jensen T.K., Jørgensen N., Swan S.H., Sapra K.J. (2016). Male Reproductive Disorders and Fertility Trends: Influences of Environment and Genetic Susceptibility. Physiol. Rev..

[141] Esteves S.C., Agarwal A. (2013). The azoospermic male: Current knowledge and future perspectives. Clinics.

[142] Tüttelmann F., Werny F., Cooper T.G., Kliesch S., Simoni M., Nieschlag E. (2011). Clinical experience with azoospermia: Aetiology and chances for spermatozoa detection upon biopsy. Int. J. Androl..

[143] Olesen I.A., Andersson A.-M., Aksglaede L., Skakkebaek N.E., Meyts E.R., Joergensen N., Juul A. (2017). Clinical, genetic, biochemical, and testicular biopsy findings among 1,213 men evaluated for infertility. Fertil. Steril..

[144] Schlegel P.N. (2004). Causes of azoospermia and their management. Reprod. Fertil. Dev..

[145] Kumar R. (2013). Medical management of non-obstructive azoospermia. Clinics.

[146] Jarvi K., Lo K., Fischer A., Grantmyre J., Zini A., Chow V., Mak V. (2010). CUA Guideline: The workup of azoospermic males. Can. Urol. Assoc. J..

[147] Berookhim B.M., Schlegel P.N. (2014). Azoospermia due to Spermatogenic Failure. Urol. Clin. N. Am..

[148] Kang C., Punjani N., Schlegel P. (2021). Reproductive Chances of Men with Azoospermia Due to Spermatogenic Dysfunction. J. Clin. Med..

[149] Fakhro K.A., Elbardisi H., Arafa M., Robay A., Rodriguez-Flores J.L., Mezey J.G., Crystal R.G., Al-Shakaki A., Syed N., Khalil C.A. (2018). Point-of-care whole-exome sequencing of idiopathic male infertility. Genet. Med..

[150] Tang Z.-R., Xu X.-L., Deng S.-L., Lian Z.-X., Yu K. (2020). Oestrogenic Endocrine Disruptors in the Placenta and the Fetus. Int. J. Mol. Sci..

[151] Kamischke A., Baumgardt A., Ju J., Horst J., Nieschlag E. (2003). Clinical and Diagnostic Features of Patients with Suspected Klinefelter Syndrome. J. Androl..

[152] Blagosklonova O., Fellmann F., Clavequin M.-C., Roux C., Bresson J.-L. (2000). AZFa deletions in Sertoli cell-only syndrome: A retrospective study. Mol. Hum. Reprod..

[153] Hopps C.V., Mielnik A., Goldstein M., Palermo G.D., Rosenwaks Z., Schlegel P.N. (2003). Detection of sperm in men with Y chromosome microdeletions of the AZFa, AZFb and AZFc regions. Hum. Reprod..

[154] Yang Y., Ma M.Y., Xiao C.Y., Li L., Li S.W., Zhang S.Z. (2008). Massive deletion in AZFb/b+c and azoospermia with Sertoli cell only and/or maturation arrest. Int J. Androl..

[155] Stahl P.J., Masson P., Mielnik A., Marean M.B., Schlegel P.N., Paduch D.A. (2010). A decade of experience emphasizes that testing for Y microdeletions is essential in American men with azoospermia and severe oligozoospermia. Fertil. Steril..

[156] Miyamoto T., Hasuike S., Yogev L., Maduro M.R., Ishikawa M., Westphal H., Lamb D.J. (2003). Azoospermia in patients heterozygous for a mutation in SYCP. Lancet.

[157] Bashamboo A., Ferraz-De-Souza B., Loureno D., Lin L., Sebire N.J., Montjean D., Bignon-Topalovic J., Mandelbaum J., Siffroi J.-P., Christin-Maitre S. (2010). Human male infertility associated with mutations in NR5A1 encoding steroidogenic factor. Am. J. Hum. Genet..

[158] Yang F., Silber S.J., Leu N.A., Oates R.D., Marszalek J.D., Skaletsky H., Brown L.G., Rozen S.G., Page D.C., Wang P.J. (2015). TEX 11 is mutated in infertile men with azoospermia and regulates genome-wide recombination rates in mouse. EMBO Mol. Med..

[159] Yatsenko A.N., Georgiadis A.P., Röpke A., Berman A.J., Jaffe T., Olszewska M., Westernströer B., Sanfilippo J., Kurpisz M., Rajkovic A. (2015). X-Linked TEX11 Mutations, Meiotic Arrest, and Azoospermia in Infertile Men. N. Engl. J. Med..

[160] Ben Khelifa M., Ghieh F., Boudjenah R., Hue C., Fauvert D., Dard R., Garchon H.J., Vialard F. (2018). A MEI1 homozygous missense mutation associated with meiotic arrest in a consanguineous family. Hum. Reprod..

[161] Yang Y., Guo J., Dai L., Zhu Y., Hu H., Tan L., Chen T., Liang D., He J., Tu M. (2018). Original article: XRCC2 mutation causes meiotic arrest, azoospermia and infertility. J. Med. Genet..

[162] Ghieh F., Mitchell V., Mandon-Pepin B., Vialard F. (2019). Genetic defects in human azoospermia. Basic Clin. Androl..

[163] Koc G., Ozdemir A.A., Girgin G., Akbal C., Kirac D., Avcilar T., Guney A.I. (2018). Male infertility in Sertoli cell-only syndrome: An investigation of autosomal gene defects. Int. J. Urol..

[164] Babakhanzadeh E., Khodadadian A., Rostami S., Alipourfard I., Aghaei M., Nazari M., Hosseinnia M., Mehrjardi M.Y.V., Jamshidi Y., Ghasemi N. (2020). Testicular expression of TDRD1, TDRD5, TDRD9 and TDRD12 in azoospermia. BMC Med. Genet..

[165] Martín M.C., Castilla J.A., Palomino-Morales R.J., Carmona F.D. (2020). Genetic Landscape of Nonobstructive Azoospermia and New Perspectives for the Clinic. J. Clin. Med..

[166] Thakker S., Persily J., Najari B.B. (2020). Kallman syndrome and central non-obstructive azoospermia. Best Pr. Res. Clin. Endocrinol. Metab..

[167] Akarsu C., Caglar G., Vicdan K., Isik A., Tuncay G. (2009). Pregnancies achieved by testicular sperm recovery in male hypogonadotrophic hypogonadism with persistent azoospermia. Reprod. Biomed. Online.

[168] Esteves S., Papanikolaou V. (2011). Clinical efficacy, safety and tolerability of recombinant human chorionic gonadotropin to restore spermatogenesis and androgen production of hypogonadotropic hypogonadal men. Fertil. Steril..

[169] Fraietta R., Zylberstejn D.S., Esteves S.C. (2013). Hypogonadotropic Hypogonadism Revisited. Clinics.

[170] Singh P., Cugati G., Singh M., Singh A.K. (2011). Hyperprolactinemia: An often missed cause of male infertility. J. Hum. Reprod. Sci..

[171] Dabbous Z., Atkin S.L. (2017). Hyperprolactinaemia in male infertility: Clinical case scenarios. Arab J. Urol..

[172] Aiman J., Griffin J.E. (1982). The Frequency of Androgen Receptor Deficiency in Infertile Men. J. Clin. Endocrinol. Metab..

[173] Akin J.W., Behzadian A., Tho S.P., McDonough P.G. (1991). Evidence for a partial deletion in the androgen receptor gene in a phenotypic male with azoospermia. Am. J. Obstet. Gynecol..

[174] Huff D.S., Fenig D.M., Canning D.A., Carr M.G., Zderic S.A., Snyder H.M. (2001). Abnormal germ cell development in cryptorchidism. Horm. Res..

[175] Sijstermans K., Hack W.W.M., Meijer R.W., Van Der Voort-Doedens L.M. (2008). The frequency of undescended testis from birth to adulthood: A review. Int. J. Androl..

[176] Fedder J. (2011). History of cryptorchidism and ejaculate volume as simple predictors for the presence of testicular sperm. Syst. Biol. Reprod. Med..

[177] Mostafa T., Anis M.T., El-Nashar A., Imam H., Othman I.A. (2001). Varicocelectomy reduces reactive oxygen species levels and increases antioxidant activity of seminal plasma from infertile men with varicocele. Int. J. Androl..

[178] Poulakis V., Ferakis N., De Vries R., Witzsch U., Becht E. (2006). Induction of spermatogenesis in men with azoospermia or severe oligoteratoasthenospermia after antegrade internal spermatic vein sclerotherapy for the treatment of varicocele. As. J. Androl..

[179] Gat Y., Gornish M., Perlow A., Chakraborty J., Levinger U., Ben-Shlomo I., Pasqualotto F. (2010). Azoospermia and Sertoli-cell-only syndrome: Hypoxia in the sperm production site due to impairment in venous drainage of male reproductive system. Andrologia.

[180] Inci K., Gunay L.M. (2013). The role of varicocele treatment in the management of non-obstructive azoospermia. Clinics.

[181] Zampieri N., Bosaro L., Costantini C., Zaffagnini S., Zampieri G. (2013). Relationship between Testicular Sperm Extraction and Varicocelectomy in Patients with Varicocele and Nonobstructive Azoospermia. Urology.

[182] Kavoussi P.K., Hunn C., Gilkey M.S., Chen S.-H., Kavoussi K.M., Wininger J.D., Kavoussi S.K. (2019). Sertoli cell only syndrome induced by a varicocele. Transl. Androl. Urol..

[183] Huyghe E., Matsuda T., Daudin M., Chevreau C., Bachaud J.M., Plante P., Bujan L., Thonneau P. (2004). Fertility after testicular cancer treatments: Results of a large multicenter study. Cancer.

[184] Gandini L., Sgrò P., Lombardo F., Paoli D., Culasso F., Toselli L., Tsamatropoulos P., Lenzi A. (2006). Effect of chemo- or radiotherapy on sperm parameters of testicular cancer patients. Hum. Reprod..

[185] Green D.M., Kawashima T., Stovall M., Leisenring W., Sklar C.A., Mertens A.C., Donaldson S.S., Byrne J., Robison L.L. (2010). Fertility of Male Survivors of Childhood Cancer: A Report from the Childhood Cancer Survivor Study. J. Clin. Oncol..

[186] Greenfield D.M., Walters S.J., Coleman R.E., Hancock B.W., Eastell R., Davies H.A., Snowden J.A., Derogatis L., Shalet S.M., Ross R.J.M. (2007). Prevalence and Consequences of Androgen Deficiency in Young Male Cancer Survivors in a Controlled Cross-Sectional Study. J. Clin. Endocrinol. Metab..

[187] Benedict C., Shuk E., Ford J.S. (2016). Fertility Issues in Adolescent and Young Adult Cancer Survivors. J. Adolesc. Young Adult Oncol..

[188] Chiba K., Yamaguchi K., Li F., Ando M., Fujisawa M. (2011). Finasteride-associated male infertility. Fertil. Steril..

[189] Samplaski M.K., Nangia A.K. (2015). Adverse effects of common medications on male fertility. Nat. Rev. Urol..

[190] Ding J., Shang X., Zhang Z., Jing H., Shao J., Fei Q., Rayburn E.R., Li H. (2017). FDA-approved medications that impair human spermatogenesis. Oncotarget.

[191] Zakhem G.A., Motosko C.C., Mu E.W., Ho R.S. (2019). Infertility and teratogenicity after paternal exposure to systemic dermatologic medications: A systematic review. J. Am. Acad. Dermatol..

[192] Bermas B.L. (2020). Paternal safety of anti-rheumatic medications. Best Pr. Res. Clin. Obstet. Gynaecol..

[193] Mylchreest E., Sar M., Wallace D.G., Foster P.M. (2002). Fetal testosterone insufficiency and abnormal proliferation of Leydig cells and gonocytes in rats exposed to di(n-butyl) phthalate. Reprod. Toxicol..

[194] Barlow N.J., Foster P.M.D. (2003). Pathogenesis of male reproductive tract lesions from gestation through adulthood following in utero exposure to Di(n-butyl) phthalate. Toxicol. Pathol..

[195] Fisher J.S., MacPherson S., Marchetti N., Sharpe R.M. (2003). Human ‘testicular dysgenesis syndrome’: A possible model using in-utero exposure of the rat to dibutyl phthalate. Hum. Reprod..

[196] Hu G.-X., Lian Q.-Q., Ge R.-S., Hardy D.O., Li X.-K. (2009). Phthalate-induced testicular dysgenesis syndrome: Leydig cell influence. Trends Endocrinol. Metab..

[197] Zhang X., Ren X., Zhang T., Zhou X., Chen X., Lu H., Zhou X., Zhang X., Wang S., Qin C. (2022). Comprehensive Analysis of the Association Between Human Non-obstructive Azoospermia and Plasticisers via Single-Cell and Traditional RNA Sequencing Methods. Expo. Health.

[198] Liu C., Duan W., Li R., Xu S., Zhang L., Chen C., He M., Lu Y., Wu H., Pi H. (2013). Exposure to bisphenol A disrupts meiotic progression during spermatogenesis in adult rats through estrogen-like activity. Cell Death Dis..

[199] Rahman M.S., Kwon W.-S., Lee J.-S., Yoon S.-J., Ryu B.-Y., Pang M.-G. (2015). Bisphenol-A Affects Male Fertility via Fertility-related Proteins in Spermatozoa. Sci. Rep..

[200] Li X., Wen Z., Wang Y., Mo J., Zhong Y., Ge R.-S. (2020). Bisphenols and Leydig Cell Development and Function. Front. Endocrinol..

[201] Pajarinen J.T., Karhunen P.J. (1994). Spermatogenic Arrest and “Sertoli Cell-Only” Syndrome--Common Alcohol-Induced Disorders of the Human Testis. Obstet. Gynecol. Surv..

[202] Sermondade N., Elloumi H., Berthaut I., Mathieu E., Delarouzière V., Ravel C., Mandelbaum J. (2010). Progressive alcohol-induced sperm alterations leading to spermatogenic arrest, which was reversed after alcohol withdrawal. Reprod. Biomed. Online.

[203] Masuda H., Inamoto T., Azuma H., Katsuoka Y., Tawara F. (2011). Successful testicular sperm extraction in an azoospermic man with postpubertal mumps orchitis. Hinyokika Kiyo. Acta Urol. J..

[204] Zhang S., An Y., Li J., Guo J., Zhou G., Li J., Xu Y. (2015). Relation between the testicular sperm assay and sex hormone level in patients with azoospermia induced by mumps. Int. J. Clin. Exp. Med..

[205] Schuppe H.-C., Pilatz A., Hossain H., Diemer T., Wagenlehner F., Weidner W. (2017). Urogenital Infection as a Risk Factor for Male Infertility. Dtsch Arztebl Int..

[206] Fijak M., Pilatz A., Hedger M.P., Nicolas N., Bhushan S., Michel V., Tung K.S.K., Schuppe H.-C., Meinhastd A. (2018). Infectious, inflammatory and ‘autoimmune’ male factor infertility: How do rodent models inform clinical practice?. Hum. Reprod..

[207] Gacci M., Coppi M., Baldi E., Sebastianelli A., Zaccaro C., Morselli S., Pecorano S., Manera A., Nicoletti S., Liaci A. (2021). Semen impairment and occurrence of SARS-CoV-2 virus in semen after recovery from COVID-19. Hum. Reprod..

[208] Hagiuda J., Ishikawa H., Hanawa Y., Marumo K. (2014). Recovery from azoospermia caused by a testicular injury: A case report. Andrologia.

[209] Alawamlh O.A.H., Flannigan R., Hayden R., Goldstein M., Li P.S., Lee R.K. (2021). Testicular Torsion and Spermatogenesis. Adv. Exp. Med. Biol..

[210] Li Z., Tian J., Cui G., Wang M., Yu D. (2015). Effects of local testicular heat treatment on Leydig cell hyperplasia and testosterone biosynthesis in rat testes. Reprod. Fertil. Dev..

[211] Ziaeipour S., Piryaei A., Aliaghaei A., Nazarian H., Naserzadeh P., Ebrahimi V., Abdi S., Shahi F., Ahmadi H., Fathabadi F.F. (2021). Chronic scrotal hyperthermia induces azoospermia and severe damage to testicular tissue in mice. Acta Histochem..

[212] Cai Z., Zhang J., Xiong J., Ma C., Yang B., Li H. (2020). New insights into the potential mechanisms of spermatogenic failure in patients with idiopathic azoospermia. Mol. Hum. Reprod..

[213] Brannigan R.E., Das A., Halpern J.A., Darves-Bornoz A.L., Patel M., Wren J., Keeter M.K. (2020). Sperm retrieval success and testicular histopathology in idiopathic nonobstructive azoospermia. As. J. Androl..

[214] Sangwan J.S., Petit C., Rose R.S., Frapsauce C., Dijols L., Rigot J.M., Guérif F. (2021). Non-obstructive idiopathic azoospermia vs azoospermia with antecedents of cryptorchidism: Ways and probabilities of becoming parents. Basic Clin. Androl..

[215] Baksi A., Vasan S.S., Dighe R.R. (2018). DNA Flow cytometric analysis of the human testicular tissues to investigate the status of spermatogenesis in azoospermic patients. Sci. Rep..

[216] Goluža T., Boscanin A., Cvetko J., Kozina V., Kosovic M., Bernat M.M., Kasum M., Kastelan Z., Jezek D. (2014). Macrophages and Leydig Cells in Testicular Biopsies of Azoospermic Men. BioMed Res. Int..

[217] Madsen A., Oehme N.B., Roelants M., Bruserud I.S., Eide G.E., Viste K., Bjerknes R., Almås B., Rosendahl K., Sagen J.V. (2020). Testicular Ultrasound to Stratify Hormone References in a Cross-Sectional Norwegian Study of Male Puberty. J. Clin. Endocrinol. Metab..

[218] Ruiz-Olvera S.F., Rajmil O., Sanchez-Curbelo J.R., Vinay J., Rodriguez-Espinosa J., Ruiz-Castañé E. (2018). Association of serum testosterone levels and testicular volume in adult patients. Andrologia.

[219] Bergmann M., Ježek D., Ježek D. (2013). Damage of Spermatogenesis. Atlas on the Human Testis.

[220] Esteves S.C., Prudencio C., Seol B., Verza S., Knoedler C., Agarwal A. (2014). Comparison of sperm retrieval and reproductive outcome in azoospermic men with testicular failure and obstructive azoospermia treated for infertility. As. J. Androl..

[221] Cito G., Coccia M.E., Picone R., Nesi G., Cocci A., Dabizzi S., Garaffa G., Fucci R., Falcone P., Bertocci F. (2018). Novel method of histopathological analysis after testicular sperm extraction in patients with nonobstructive and obstructive azoospermia. Clin. Exp. Reprod. Med..

[222] Toksöz S., Kizilkan Y. (2019). Comparison of the Histopathological Findings of Testis Tissues of Non-Obstructive Azoospermia with the Findings after Microscopic Testicular Sperm Extraction. Urol. J..

[223] Nistal M., Paniagua R., Riestra M.L., Reyes-Múgica M., Cajaiba M.M. (2007). Bilateral prepubertal testicular biopsies predict significance of cryptorchidism-associated mixed testicular atrophy, and allow assessment of fertility. Am. J. Surg. Pathol..

[224] Van Casteren N.J., Looijenga L.H.J., Dohle G.R. (2009). Testicular microlithiasis and carcinoma in situ overview and proposed clinical guideline. Int. J. Androl..

[225] Barbonetti A., Martorella A., Minaldi E., D’Andrea S., Bardhi D., Castellini C., Francavilla F., Francavilla S. (2019). Testicular Cancer in Infertile Men with and Without Testicular Microlithiasis: A Systematic Review and Meta-Analysis of Case-Control Studies. Front. Endocrinol..

[226] Eisenberg M.L., Betts P., Herder D., Lamb D.J., Lipshultz L.I. (2013). Increased risk of cancer among azoospermic men. Fertil. Steril..

[227] Bay K., Asklund C., Skakkebaek N.E., Andersson A.-M. (2006). Testicular dysgenesis syndrome: Possible role of endocrine disrupters. Best Pr. Res. Clin. Endocrinol. Metab..

[228] Slowikowska-Hilczer J., Szarras-Czapnik M., Wolski J.K., Oszukowska E., Hilczer M., Jakubowski L., Walczak-Jedrzejowska R., Marchlewska K., Filipiak E., Kaluzewski B. (2015). The risk of neoplasm associated with dysgenetic testes in prepubertal and pubertal/adult patients. Folia Histochem. Cytobiol..

[229] Niedzielski J.K., Oszukowska E., Słowikowska-Hilczer J. (2016). Undescended testis—Current trends and guidelines: A review of the literature. Arch. Med. Sci..

[230] Sato Y., Nozawa S., Iwamoto T. (2008). Study of spermatogenesis and thickening of lamina propria in the human seminiferous tubules. Fertil. Steril..

[231] Volkmann J., Muller D., Feuerstacke C., Kliesch S., Bergmann M., Mühlfeld C., Middendorff R. (2011). Disturbed spermatogenesis associated with thickened lamina propria of seminiferous tubules is not caused by dedifferentiation of myofibroblasts. Hum. Reprod..

[232] Sato Y., Nozawa S., Yoshiike M., Otoi T., Iwamoto T. (2012). Glycoconjugates recognized by peanut agglutinin lectin in the inner acellular layer of the lamina propria of seminiferous tubules in human testes showing impaired spermatogenesis. Hum. Reprod..

[233] Ooba T., Ishikawa T., Yamaguchi K., Kondo Y., Sakamoto Y., Fujisawa M. (2008). Expression and Distribution of Laminin Chains in the Testis for Patients with Azoospermia. J. Androl..

[234] Oka S., Shiraishi K., Matsuyama H. (2017). Effects of human chorionic gonadotropin on testicular interstitial tissues in men with non-obstructive azoospermia. Andrology.

[235] Mahran A.M., Elgamal D.A., Ghafeer H.H., Abdel-Maksoud S.A., Farrag A.A. (2016). Histological alterations in Leydig cells and macrophages in azoospermic men. Andrologia.

[236] Ježek D., Knežević N., Banek L., Krhen I., Muzic V., Kalanj-Bognar S., Sincic S., Zimak Z., Kastelan Z., Jezej V. (2002). Fine Structure of Leydig Cells in Patients with Non-Obstructive Azoospermia. Acta Clin. Croat..

[237] Carucci L.R., Tirkes A.T., Pretorius E.S., Genega E.M., Weinstein S.P. (2003). Testicular Leydig’s Cell Hyperplasia: MR Imaging and Sonographic Findings. AJR Am. J. Roentgenol..

[238] Li X., Strauss L., Kaatrasalo A., Mayerhofer A., Huhtaniemi I., Santti R., Mäkelä S., Poutanen M. (2006). Transgenic Mice Expressing P450 Aromatase as a Model for Male Infertility Associated with Chronic Inflammation in the Testis. Endocrinology.

[239] Li X., Li H., Jia L., Li X., Rahman N. (2015). Oestrogen action and male fertility: Experimental and clinical findings. Cell Mol. Life Scixp..

[240] Aksglæde L., Skakkebæk N.E., Almstrup K., Juul A. (2011). Clinical and biological parameters in 166 boys, adolescents and adults with nonmosaic Klinefelter syndrome: A Copenhagen experience. Acta Paediatr..

[241] Yu W., Zheng H., Lin W., Tajima A., Zhang Y., Zhang X., Zhang H., Wu J., Han D., Rahman N.A. (2014). Estrogen promotes Leydig cell engulfment by macrophages in male infertility. J. Clin. Investig..

[242] Newman C., Connolly S., MacEneaney O., O’Keane C., McQuaid S.E. (2019). Leydig Cell Hyperplasia Mimicking a Testicular Tumour in a Patient with Klinefelter Syndrome. Eur. J. Case Rep. Intern. Med..

[243] Tash J.A., McCallum S., Hardy M.P., Knudsen B., Schlegel P.N. (2002). Men with nonobstructive azoospermia have Leydig cell hypertrophy but not hyperplasia. J. Urol..

[244] McKinnell C., Sharpe R.M., Mahood K., Hallmark N., Scott H., Ivell R., Staub C., Jegou B., Haag F., Koch-Nolte F. (2005). Expression of Insulin-Like Factor 3 Protein in the Rat Testis during Fetal and Postnatal Development and in Relation to Cryptorchidism Induced by in Utero Exposure to Di (n-Butyl) Phthalate. Endocrinology.

[245] Lin H., Ge R.-S., Chen G.-R., Hu G.-X., Dong L., Lian Q.-Q., Hardy D.O., Sottas C.M., Li X.-K., Hardy M.P. (2008). Involvement of testicular growth factors in fetal Leydig cell aggregation after exposure to phthalate *in utero*. Proc. Natl. Acad. Sci. USA.

[246] Hauptman D., Perić M.H., Marić T., Bojanac A.K., Sinčić N., Zimak Z., Kastelan Z., Jezevk D. (2021). Leydig Cells in Patients with Non-Obstructive Azoospermia: Do They Really Proliferate?. Life.

[247] Shono T., Suita S. (2009). Reasonable explanation for both the antiandrogenic mechanism of DBP and DBP-induced Leydig cell hyperplasia in prenatally DBP-treated rats. Toxicol. Appl. Pharmacol..

[248] Mahood I.K., Hallmark N., McKinnell C., Walker M., Fisher J.S., Sharpe R.M. (2005). Abnormal Leydig Cell Aggregation in the Fetal Testis of Rats Exposed to Di (n-Butyl) Phthalate and Its Possible Role in Testicular Dysgenesis. Endocrinology.

[249] Winge S.B., Dalgaard M.D., Belling K.G., Jensen J.M., Nielsen J.E., Aksglaede L., Schierup M.H., Brunak S., Skakkebæk N.E., Juul A. (2018). Transcriptome analysis of the adult human Klinefelter testis and cellularity-matched controls reveals disturbed differentiation of Sertoli- and Leydig cells. Cell Death Dis..

[250] Welsh M., Moffat L., Belling K., de França L.R., Segatelli T.M., Saunders P.T.K., Sharpe R.M., Smith L.B. (2012). Androgen receptor signalling in peritubular myoid cells is essential for normal differentiation and function of adult Leydig cells. Int. J. Androl..

[251] Tanrikut C., Goldstein M., Rosoff J.S., Lee R.K., Nelson C.J., Mulhall J.P. (2011). Varicocele as a risk factor for androgen deficiency and effect of repair. Br. J. Urol..

[252] Abdel-Razic M.M., Abdel-Hamid I.A., Elsobky E., El-Dahtory F. (2012). Further Evidence of the Clinical, Hormonal, and Genetic Heterogeneity of Klinefelter Syndrome: A Study of 216 Infertile Egyptian Patients. J. Androl..

[253] Bobjer J., Naumovska M., Giwercman Y., Giwercman A. (2012). High prevalence of androgen deficiency and abnormal lipid profile in infertile men with non-obstructive azoospermia. Int. J. Androl..

[254] Rohayem J., Luberto A., Nieschlag E., Zitzmann M., Kliesch S. (2017). Delayed treatment of undescended testes may promote hypogonadism and infertility. Endocrine.

[255] Isaksson S., Bogefors K., Ståhl O., Eberhard J., Giwercman Y., Leijonhufvud I., Link K., Øra I., Romerius P., Bobjer J. (2018). High risk of hypogonadism in young male cancer survivors. Clin. Endocrinol..

[256] Ma D., Luo N., Xue G. (2019). Trimethyltin (TMT) Reduces Testosterone Production in Adult Leydig Cells in Rats. Int. J. Toxicol..

[257] Akingbemi B.T., Sottas C.M., Koulova A.I., Klinefelter G.R., Hardy M.P. (2004). Inhibition of Testicular Steroidogenesis by the Xenoestrogen Bisphenol A Is Associated with Reduced Pituitary Luteinizing Hormone Secretion and Decreased Steroidogenic Enzyme Gene Expression in Rat Leydig Cells. Endocrinology.

[258] Welsh M., Saunders P.T., Fisken M., Scott H.M., Hutchison G.R., Smith L.B., Sharpe R.M. (2008). Identification in rats of a programming window for reproductive tract masculinization, disruption of which leads to hypospadias and cryptorchidism. J. Clin. Investig..

[259] van den Driesche S., Scott H.M., MacLeod D.J., Fisken M., Walker M., Sharpe R.M. (2011). Relative importance of prenatal and postnatal androgen action in determining growth of the penis and anogenital distance in the rat before, during and after puberty. Int. J. Androl..

[260] Sato Y., Asahina K., Yoshiike M., Nozawa S., Otoi T., Iwamoto T. (2020). A change in the steroid metabolic pathway in human testes showing deteriorated spermatogenesis. Reprod. Biol..

[261] Abid S., Maitra A., Meherji P., Patel Z., Kadam S., Shah J., Shah R., Kulkarni V., Baburao V., Gokral J. (2008). Clinical and laboratory evaluation of idiopathic male infertility in a secondary referral center in India. J. Clin. Lab. Anal..

[262] Koşar P.A., Özçelik N., Koşar A. (2010). Cytogenetic abnormalities detected in patients with non-obstructive azoospermia and severe oligozoospermia. J. Assist. Reprod. Genet..

[263] Ekman B., Fitts D., Marelli C., Murray R.D., Quinkler M., Zelissen P.M. (2014). European Adrenal Insufficiency Registry (EU-AIR): A comparative observational study of glucocorticoid replacement therapy. BMC Endocr. Disord..

[264] Fukuda I., Hizuka N., Muraoka T., Ichihara A. (2014). Adult Growth Hormone Deficiency: Current Concepts. Neurol. Med.-Chirurg..

[265] Gámez J.M., Penalba R., Cardoso N., Ponzo O., Carbone S., Pandolfi M., Scacchi P., Reynoso R. (2013). Low dose of bisphenol A impairs the reproductive axis of prepuberal male rats. J. Physiol. Biochem..

[266] Bahmanimehr A., Zeighami S., Jahromi B.N., Parsanezhad M.E., Davari M., Montazeri S., Vaziri N.M., Zarei A. (2018). Detection of Y Chromosome Microdeletions and Hormonal Profile Analysis of Infertile Men undergoing Assisted Reproductive Technologies. Int. J. Fertil. Steril..

[267] Johnson M., Raheem A., De Luca F., Hallerstrom M., Zainal Y., Poselay S., Mohammadi B., Moubasher A., Johnson T.F., Muneer A. (2019). An analysis of the frequency of Y-chromosome microdeletions and the determination of a threshold sperm concentration for genetic testing in infertile men. Br. J. Urol..

[268] Jørgensen N., Joensen U.N., Toppari J., Punab M., Erenpreiss J., Zilaitiene B., Paasch U., Salzbrunn A., Fernandez M.F., Virtanen H. (2016). Compensated reduction in Leydig cell function is associated with lower semen quality variables: A study of 8182 European young men. Hum. Reprod..

[269] Bandak M., Jørgensen N., Juul A., Lauritsen J., Oturai P., Mortensen J., Hojman P., Helge J., Daugaard G. (2017). Leydig cell dysfunction, systemic inflammation and metabolic syndrome in long-term testicular cancer survivors. Eur. J. Cancer.

[270] Johnston H., Baker P.J., Abel M., Charlton H.M., Jackson G., Fleming L., Kumar T.R., O’Shaughnessy P.J. (2004). Regulation of Sertoli Cell Number and Activity by Follicle-Stimulating Hormone and Androgen during Postnatal Development in the Mouse. Endocrinology.

[271] Suomi A.-M., Main K.M., Kaleva M., Schmidt I.M., Chellakooty M., Virtanen H.E., Boisen K.A., Damgaard I.N., Kai C.M., Skakkebæk N.E. (2006). Hormonal Changes in 3-Month-Old Cryptorchid Boys. J. Clin. Endocrinol. Metab..

[272] Skøtt J.W., Lauritsen J., Kreiberg M., Daugaard G., Bandak M. (2019). Quality of Life in Long-Term Testicular Cancer Survivors with Compensated Leydig Cell Dysfunction. Clin. Genitourin. Cancer.

[273] Lardone M.C., Castillo P., Valdevenito R., Ebensperger M., Ronco A.M., Pommer R., Piottante A., Castro A. (2010). P450-aromatase activity and expression in human testicular tissues with severe spermatogenic failure. Int. J. Androl..

[274] Tarsitano M.G., Bandak M., Jørgensen N., Skakkebæk N.E., Juul A., Lenzi A., Daugaard D., Rajpert-De Meyts E. (2018). Quantification of the Leydig cell compartment in testicular biopsies and association with biochemical Leydig cell dysfunction in testicular cancer survivors. Andrology.

[275] Akingbemi B.T., Ge R., Klinefelter G.R., Zirkin B.R., Hardy M.P. (2004). Phthalate-induced Leydig cell hyperplasia is associated with multiple endocrine disturbances. Proc. Natl. Acad. Sci. USA.

[276] Akingbemi B.T., Braden T.D., Kemppainen B.W., Hancock K.D., Sherrill J.D., Cook S.J., He X., Supko J.G. (2007). Exposure to Phytoestrogens in the Perinatal Period Affects Androgen Secretion by Testicular Leydig Cells in the Adult Rat. Endocrinology.

[277] Shiraishi K., Oka S., Matsuyama H. (2020). Testicular Testosterone and Estradiol Concentrations and Aromatase Expression in Men with Nonobstructive Azoospermia. J. Clin. Endocrinol. Metab..

[278] Strauss L., Kallio J., Desai N., Pakarinen P., Miettinen T., Gylling H., Albercht M., Makela S., Mayerhofer A., Poutanen M. (2009). Increased Exposure to Estrogens Disturbs Maturation, Steroidogenesis, and Cholesterol Homeostasis via Estrogen Receptor α in Adult Mouse Leydig Cells. Endocrinology.

[279] Lardone M.C., Argandoña F., Flórez M., Parada-Bustamante A., Ebensperger M., Palma C., Piottante A., Castro A. (2017). Overexpression of CYP19A1 aromatase in Leydig cells is associated with steroidogenic dysfunction in subjects with Sertoli cell-only syndrome. Andrology.

[280] Lardone M.C., Reyes I.N., Ortiz E., Piottante A., Palma C., Ebensperger M., Castro A. (2021). Testicular steroid sulfatase overexpression is associated with Leydig cell dysfunction in primary spermatogenic failure. Andrology.

[281] Lardone M.C., Argandoña F., Lorca M., Piottante A., Flórez M., Palma C., Ebensperger E., Castro A. (2018). Leydig cell dysfunction is associated with post-transcriptional deregulation of CYP17A1 in men with Sertoli cell-only syndrome. Mol. Hum. Reprod..

[282] Marsh C.A., Auchus R.J. (2014). Fertility in patients with genetic deficiencies of cytochrome P450c17 (CYP17A1): Combined 17-hydroxylase/17,20-lyase deficiency and isolated 17,20-lyase deficiency. Fertil. Steril..

[283] Luboshitzky R., Kaplan-Zverling M., Shen-Orr Z., Nave R., Herer P. (2002). Seminal plasma androgen/oestrogen balance in infertile men. Int. J. Androl..

[284] McKinnell C., Atanassova N., Williams K., Fisher J.S., Walker M., Turner K.J., Saunders T.K., Sharpe R.M. (2001). Suppression of androgen action and the induction of gross abnormalities of the reproductive tract in male rats treated neonatally with diethylstilbestrol. J. Androl..

[285] Rivas A., Fisher J.S., McKinnell C., Atanassova N., Sharpe R.M. (2002). Induction of Reproductive Tract Developmental Abnormalities in the Male Rat by Lowering Androgen Production or Action in Combination with a Low Dose of Diethylstilbestrol: Evidence for Importance of the Androgen-Estrogen Balance. Endocrinology.

[286] Han Y., Feng H.L., Sandlow J.I., Haines C.J. (2009). Comparing Expression of Progesterone and Estrogen Receptors in Testicular Tissue from Men With Obstructive and Nonobstructive Azoospermia. J. Androl..

[287] Mizuno K., Kojima Y., Kurokawa S., Kamisawa H., Kohri K., Hayashi Y. (2011). Altered Expression and Localization of Estrogen Receptors Alpha and Beta in the Testes of a Cryptorchid Rat Model. Urology.

[288] Sansone A., Kliesch S., Isidori A.M., Schlatt S. (2019). AMH and INSL3 in testicular and extragonadal pathophysiology: What do we know?. Andrology.

[289] Overvad S., Bay K., Bojesen A., Gravholt C.H. (2014). Low INSL3 in Klinefelter syndrome is related to osteocalcin, testosterone treatment and body composition, as well as measures of the hypothalamic-pituitary-gonadal axis. Andrology.

[290] Di Nisio A., De Toni L., Rocca M.S., Ghezzi M., Selice R., Taglialavoro G., Ferlin A., Forestra C. (2018). Negative Association Between Sclerostin and INSL3 in Isolated Human Osteocytes and in Klinefelter Syndrome: New Hints for Testis–Bone Crosstalk. J. Clin. Endocrinol. Metab..

[291] Trabado S., Maione L., Bry-Gauillard H., Affres H., Salenave S., Sarfati J., Bouvattier C., Delemer B., Chanson P., Le Bouc Y. (2014). Insulin-like Peptide 3 (INSL3) in Men with Congenital Hypogonadotropic Hypogonadism/Kallmann Syndrome and Effects of Different Modalities of Hormonal Treatment: A Single-Center Study of 281 Patients. J. Clin. Endocrinol. Metab..

[292] Feng S., Cortessis V.K., Hwang A., Hardy B., Koh C.J., Bogatcheva N., Agoulnik A.I. (2004). Mutation analysis of INSL3 and GREAT/LGR8 genes in familial cryptorchidism. Urology.

[293] Foresta C., Ferlin A. (2004). Role of INSL3 and LGR8 in cryptorchidism and testicular functions. Reprod. Biomed. Online.

[294] Ayers K., Kumar R., Robevska G., Bruell S., Bell K., Malik M.A., Bathgate R., Sinclair A. (2019). Familial bilateral cryptorchidism is caused by recessive variants in RXFP. J. Med. Genet..

[295] Bay K., Andersson A.-M. (2010). Human testicular insulin-like factor 3: In relation to development, reproductive hormones and andrological disorders. Int. J. Androl..

[296] Lee S.-Y., Park E., Kim S.-C., Ahn R.-S., Ko C., Lee K. (2012). ERα/E2 signaling suppresses the expression of steroidogenic enzyme genes via cross-talk with orphan nuclear receptor Nur77 in the testes. Mol. Cell. Endocrinol..

[297] Laguë E., Tremblay J.J. (2008). Antagonistic Effects of Testosterone and the Endocrine Disruptor Mono-(2-Ethylhexyl) Phthalate on INSL3 Transcription in Leydig Cells. Endocrinology.

[298] Laguë A., Tremblay J.J. (2009). Estradiol represses Insulin-like 3 expression and promoter activity in MA-10 Leydig cells. Toxicology.

[299] Soerensen R.R., Johannsen T.H., Skakkebaek N.E., Meyts E.R.-D. (2016). Leydig cell clustering and Reinke crystal distribution in relation to hormonal function in adult patients with testicular dysgenesis syndrome (TDS) including cryptorchidism. Hormones.

[300] Wohlfahrt-Veje C., Main K.M., Skakkebæk N.E. (2009). Testicular dysgenesis syndrome: Foetal origin of adult reproductive problems. Clin. Endocrinol..

[301] Olesen I.A., Joensen U.N., Petersen J.H., Almstrup K., Rajpert-De Meyts E., Carlsen E., McLachlan R., Juul A., Jørgensen N. (2018). Decrease in semen quality and Leydig cell function in infertile men: A longitudinal study. Hum. Reprod..

[302] Sharpe R.M., Skakkebaek N.E. (2003). Male reproductive disorders and the role of endocrine disruption: Advances in understanding and identification of areas for future research. Pure Appl. Chem..

[303] O’Donnell L., Robertson K.M., Jones M.E., Simpson E.R. (2001). Estrogen and Spermatogenesis. Endocr. Rev..

